# Genotypic and phenotypic diversity among *Komagataella* species reveals a hidden pathway for xylose utilization

**DOI:** 10.1186/s12934-022-01796-3

**Published:** 2022-04-25

**Authors:** Lina Heistinger, Juliane C. Dohm, Barbara G. Paes, Daniel Koizar, Christina Troyer, Özge Ata, Teresa Steininger-Mairinger, Diethard Mattanovich

**Affiliations:** 1 Department of Biotechnology, Institute of Microbiology and Microbial Biotechnology, University of Natural Resources and Life Sciences Vienna (BOKU), 1190 Vienna, Austria; 2 Department of Biotechnology, Institute of Computational Biology, University of Natural Resources and Life Sciences Vienna (BOKU), 1190 Vienna, Austria; 3 Department of Cell Biology, Institute of Biological Sciences, University of Brasilia (UnB), Brasilia, Brazil; 4 Department of Chemistry, Institute of Analytical Chemistry, University of Natural Resources and Life Sciences Vienna (BOKU), 1190 Vienna, Austria; 5 Austrian Centre of Industrial Biotechnology (Acib GmbH), 1190 Vienna, Austria; 6 Present Address: Institute of Biochemistry, Department of Biology, ETH Zürich, 8093 Zürich, Switzerland

**Keywords:** *Komagataella* species, *Pichia pastoris*, Xylose assimilation, Yeast diversity, Genome sequencing

## Abstract

**Background:**

The yeast genus *Komagataella* currently consists of seven methylotrophic species isolated from tree environments. Well-characterized strains of *K. phaffii* and *K. pastoris* are important hosts for biotechnological applications, but the potential of other species from the genus remains largely unexplored. In this study, we characterized 25 natural isolates from all seven described *Komagataella* species to identify interesting traits and provide a comprehensive overview of the genotypic and phenotypic diversity available within this genus.

**Results:**

Growth tests on different carbon sources and in the presence of stressors at two different temperatures allowed us to identify strains with differences in tolerance to high pH, high temperature, and growth on xylose. As *Komagataella* species are generally not considered xylose-utilizing yeasts, xylose assimilation was characterized in detail. Growth assays, enzyme activity measurements and ^13^C labeling confirmed the ability of *K. phaffii* to utilize D-xylose via the oxidoreductase pathway. In addition, we performed long-read whole-genome sequencing to generate genome assemblies of all *Komagataella* species type strains and additional *K. phaffii* and *K. pastoris* isolates for comparative analysis. All sequenced genomes have a similar size and share 83–99% average sequence identity. Genome structure analysis showed that *K. pastoris* and *K. ulmi* share the same rearrangements in difference to *K. phaffii*, while the genome structure of *K. kurtzmanii* is similar to *K. phaffii*. The genomes of the other, more distant species showed a larger number of structural differences. Moreover, we used the newly assembled genomes to identify putative orthologs of important xylose-related genes in the different *Komagataella* species.

**Conclusions:**

By characterizing the phenotypes of 25 natural *Komagataella* isolates, we could identify strains with improved growth on different relevant carbon sources and stress conditions. Our data on the phenotypic and genotypic diversity will provide the basis for the use of so-far neglected *Komagataella* strains with interesting characteristics and the elucidation of the genetic determinants of improved growth and stress tolerance for targeted strain improvement.

**Supplementary Information:**

The online version contains supplementary material available at 10.1186/s12934-022-01796-3.

## Introduction

Yeasts are unicellular fungi naturally occurring in a wide variety of different habitats. More than 1500 species with high phylogenetic diversity are known so far. Several of ascomycete yeast species are very well studied, of which many are used in the food industry and for biotechnological applications. However, most isolated species have not been thoroughly characterized so far. Analysis of genome sequences and phenotypic traits of these yeasts will help to understand the evolution of yeast species and provide a great resource for industrial strain development.

The yeast genus *Komagataella* consists of seven methylotrophic species classified according to the divergence of marker gene sequences [1]. All the available strains were isolated from tree environments in Europe and North America. So far, only strains of two *Komagataella* species, *K. phaffii* and *K. pastoris* (both formerly classified as *Pichia pastoris*), have been studied in detail. Originally investigated for the production of single-cell protein from methanol, strains of *K. phaffii* and *K. pastoris* are now mostly used for recombinant protein production. Complete genome assemblies and gene annotations have been published for *K. phaffii* CBS 7435, its histidine auxotrophic derivative GS115, an *AOX1* deletion (mutS) strain, and the *K. pastoris* type strain CBS 704 [2–6]. More recently, the genome sequencing data of six additional natural *K. phaffii* isolates, including the CBS 2612 type strain, have become available and up to 44,000 single nucleotide polymorphisms (SNPs) could be identified between some of the strains [7, 8]. Also, several transcriptomics and proteomics studies under different industrially relevant conditions have been performed [9], which enables targeted genome engineering for improved recombinant protein production and metabolic engineering. However, there is little information available on strains of other *Komagataella* species.

As methylotrophic yeasts, *Komagataella* species can utilize methanol as a carbon and energy source, and they show fast growth to high cell densities on carbon sources like glucose, glycerol, ethanol, and methanol under aerobic conditions. Usually, cells are grown at temperatures of 25–30 °C, but growth between 20 and 37 °C is possible*. K. phaffii* strains can tolerate neutral to acidic pH down to pH 3 [10], while the pH is usually kept at 5–6 during fermentation processes.

*Komagataella* species are generally not known to assimilate the pentose sugar d-xylose, although one study reported slow growth of non-engineered *K. phaffii* GS115 cells [11]. Also, the formation of xylitol from xylose without visible cell growth has been described for *K. pastoris* [12]. Xylose is highly abundant in lignocellulosic biomass (around 30% of total carbohydrate monomers), which makes it a promising renewable resource for biotechnological processes. Xylose can be utilized by different species of bacteria, archaea, yeast and filamentous fungi. Identification of robust xylose utilizing species and genetic engineering of xylose-fermenting yeasts is highly relevant for industrial ethanol and chemical production [13–15]. In efficient xylose-utilizing yeast species like *Scheffersomyces stipitis* and *Sugiyamaella lignohabitans*, xylose assimilation takes place via an oxidoreductase pathway. In the first step, xylose is reduced to xylitol by xylose reductase (XR), which requires NADPH or NADH as cofactor [16]. Subsequently, xylitol is converted to xylulose by xylitol dehydrogenase (XDH) using NAD^+^ [17]. As a result, xylose reductases preferentially using NADPH as cofactor can cause cofactor imbalance and carbon loss through excess xylitol formation [18]. In the next step, xylulose is converted to xylulose-5-phosphate by xylulokinase (XK) using ATP and is further metabolized via the pentose phosphate pathway [13, 19]. Compared to other carbon sources, xylose is fermented by only few described yeast species. However, many more species have been shown to grow on xylose as carbon source or have putative xylose assimilation pathway genes [20, 21].

We present a comparative characterization of the phenotypic and genotypic diversity of 25 natural isolates from all seven known *Komagataella* species. Differences in cell growth were assessed on different carbon sources and in the presence of stressors at two different temperatures. In addition, we performed a detailed characterization of xylose assimilation, including ^13^C labeling. To assess the genotypic diversity within the genus, the genomes of the type strains of *K. pseudopastoris*, *K. populi*, *K. kurtzmanii*, *K. ulmi* and *K. mondaviorum*, as well as additional *K. phaffii* and *K. pastoris* strains were sequenced on the PacBio platform and used for comparative analysis of genome structure and sequence diversity.

## Results

### Genome sequencing of all *Komagataella* species

We generated whole-genome sequencing data for all seven species of the genus *Komagataella* using the Pacific Biosciences (PacBio) technology resulting in 7.9 × 10^6^ subreads (43.8 Gbp) with read- lengths between ~ 1 kbp and 20 kbp (Additional file 1: Figure S1). For five species (*K. kurtzmanii*, *K. mondaviorum*, *K. populi*, *K. pseudopastoris*, and *K. ulmi*) the type strains were sequenced. Genome assemblies of the remaining two species, *K. pastoris* type strain CBS 704 and *K. phaffii* CBS 7435, which was recently shown to be highly similar to the type strain *K. phaffii* CBS 2612, were already available [4, 5, 7, 8]. Therefore, we selected additional strains of these species for sequencing. In total, eight strains were sequenced and assembled (two strains of *K. pastoris*, one strain for each of the other species). The quality of our assemblies was comparable to the two available assemblies with an average N50 size of 2.34 Mbp (Table 1).

**Table 1:** Assembly metrics for eight *Komagataella* strains assembled using PacBio whole-genome sequencing reads before removal of mitochondrial sequences

	Total assembly size (Mbp)	Number of contigs	N50 size (Mbp)	N50 number	Longest sequence (Mbp)	Sum of four largest sequences (Mbp)
*K. mondaviorum* CBS 15017	9.5	13	2.38	2	3.15	8.5
*K. ulmi* CBS 12361	9.6	11	2.74	2	3.30	9.5
*K. populi* CBS 12362	9.3	11	2.61	2	2.80	8.4
*K. kurtzmanii* CBS 12817	9.6	19	1.67	3	2.42	7.5
*K. phaffii* UWOPS 03-328y3	9.5	8	2.27	2	2.90	8.6
*K. pastoris* DSMZ 70877	9.6	8	2.72	2	3.32	9.5
*K. pastoris* CBS 9178	10.1	34	2.69	2	3.32	9.4
*K. pseudopastoris* CBS 9187	10.6	73	1.67	3	2.80	7.9
Ref. *K. pastoris* CBS 704	9.6	11	2.69	2	3.33	9.4
Ref. *K. phaffii* CBS 7435	9.4	7	2.40	2	2.89	9.4

For comparison, the two existing reference assemblies of *K. pastoris* (ASM170810v1) and *K. phaffii* (ASM170808v1) were added (indicated by “Ref.”)

### Phylogeny based on whole-genome sequencing data

**Fig. 1:**
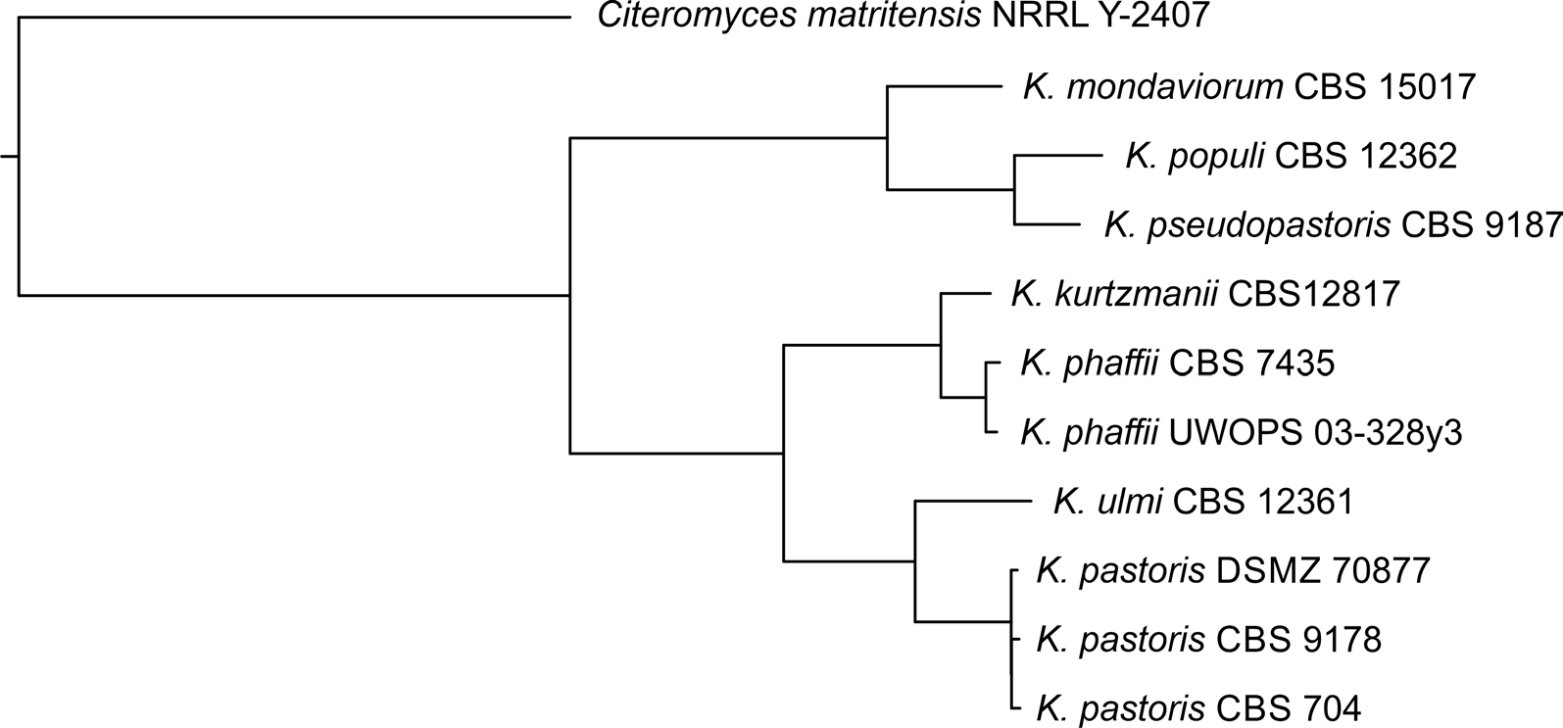
Phylogenetic tree of *Komagataella* species. The tree was calculated based on pairwise genomic distances between the assembled genomes as determined by Mash [22]. The genome sequence of *Citeromyces matritensis* NRRL Y-2407 (NCBI id ASM370516v1) was used as outgroup

The phylogenetic relationship of the seven *Komagataella* species was determined based on pairwise comparisons of a representative selection of subsequences from each genome assembly (resulting in “Mash” distances, Additional file 1: Table S1). In total, eleven genomes were included in the analysis, i.e. eight genomes assembled by us (see above), the two publicly available genomes of *K. phaffii* and *K. pastoris*, and the genome of *Citeromyces matritensis* [21] as outgroup. In the phylogenetic tree based on Mash distances the three *K. pastoris* strains clustered together, as well as the two *K. phaffii* strains (Fig. 1). The tree showed the relationship of *K. ulmi* to *K. pastoris* and of *K. kurtzmanii* to *K. phaffii* in two subtrees that were placed as sister groups, and the relationship of the species *K. mondaviorum*, *K. populi*, and *K. pseudopastoris* grouping together as a separate clade. The tree topology confirmed the findings of a previous study based on selected marker genes [1].

### Divergence in the genome structure of *Komagataella* species

**Fig. 2:**
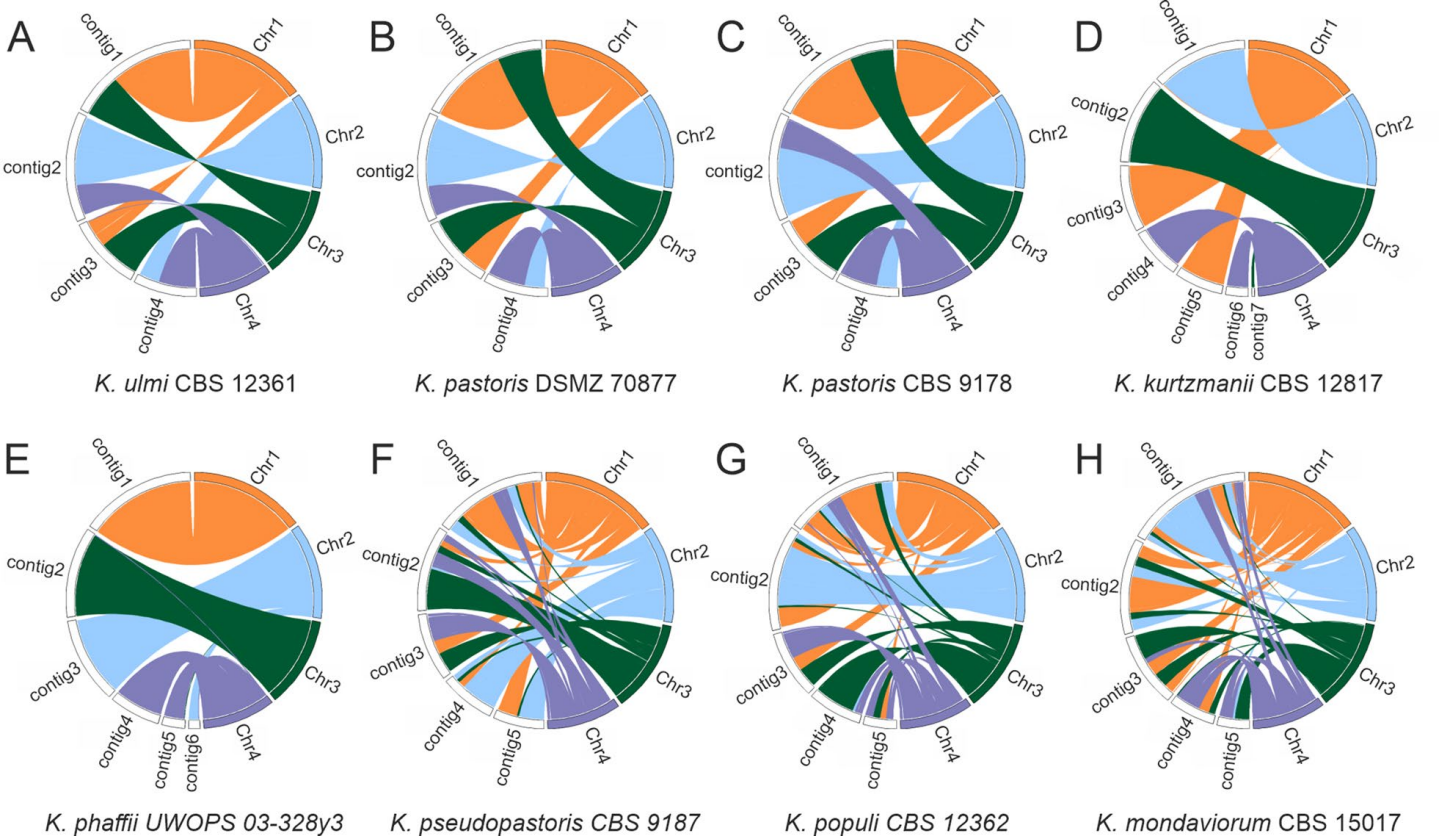
Regions of similarity between the reference strain *K. phaffii* CBS 7435 (NCBI assembly id ASM170808v1) and *K. ulmi* CBS 12361 (**A**), *K. pastoris* DSMZ 70877 (**B**), *K. pastoris* CBS 9178 (**C**), *K. kurtzmanii* CBS 12817 (**D**), *K. phaffii* UWOPS 03-328y3 (**E**), *K. pseudopastoris* CBS 9187 (**F**), *K. populi* CBS 12362 (**G**) and *K. mondaviorum* CBS 15017 (**H**) based on whole-genome comparisons. Colors refer to the four chromosomes (chr1: orange, chr2: blue, chr3: green, chr4: purple), contigs appear in their initial order and orientation of the assemblies. Only matches spanning at least 10 kbp are shown and non-matching contigs were removed

Previous genome comparisons between *K. phaffii* and *K. pastoris* revealed genomic rearrangements [5, 23] which can be summarised as one exchange between chromosomes 1 and 3 and another one between chromosomes 2 and 4. We compared our genome assemblies with *K. phaffii* CBS 7435 as reference and detected the same pattern in *K. ulmi* (genome structure similar to *K. pastoris*) and *K. kurtzmanii* (genome structure similar to *K. phaffii*) consistent with the placement in the phylogenetic tree (Fig. 2A–E). The method to determine genomic distances for tree calculation takes short subsequences into account rather than large genomic regions, so that the divergence between the two clades was demonstrated both on the sequence level and in the genome structure.

Based on these genome comparisons and available chromosome information [5] we inferred the contig order and orientation for the five genome assemblies including chromosomal assignment (Table 2). The reference assemblies used for comparison consisted of four sequences (manually connected contigs, see [5]) corresponding to four chromosomes.

**Table 2:** Chromosome assignment, order, and orientation of contigs based on whole-genome comparisons to *K. phaffii* CBS 7435 and available chromosome information [5]

	Chr1	Chr2	Chr3	Chr4
*K. ulmi* CBS 12361	contig1	contig2^a^	contig3	contig4
*K. pastoris* DSMZ 70877	contig1^a^	contig2^a^	contig3^a^	contig4^a^
*K. pastoris* CBS 9178	contig1^a^	contig2	contig3	contig4^a^
*K. kurtzmanii* CBS 12817	contig5 contig3	contig1^a^	contig2 contig7	contig4^a^ contig6
*K. phaffii* UWOPS 03-328y3	contig1	contig3 contig6	contig2	contig5 contig4

^a^Contig in reverse orientation compared to the reference chromosome

**Fig. 3:**
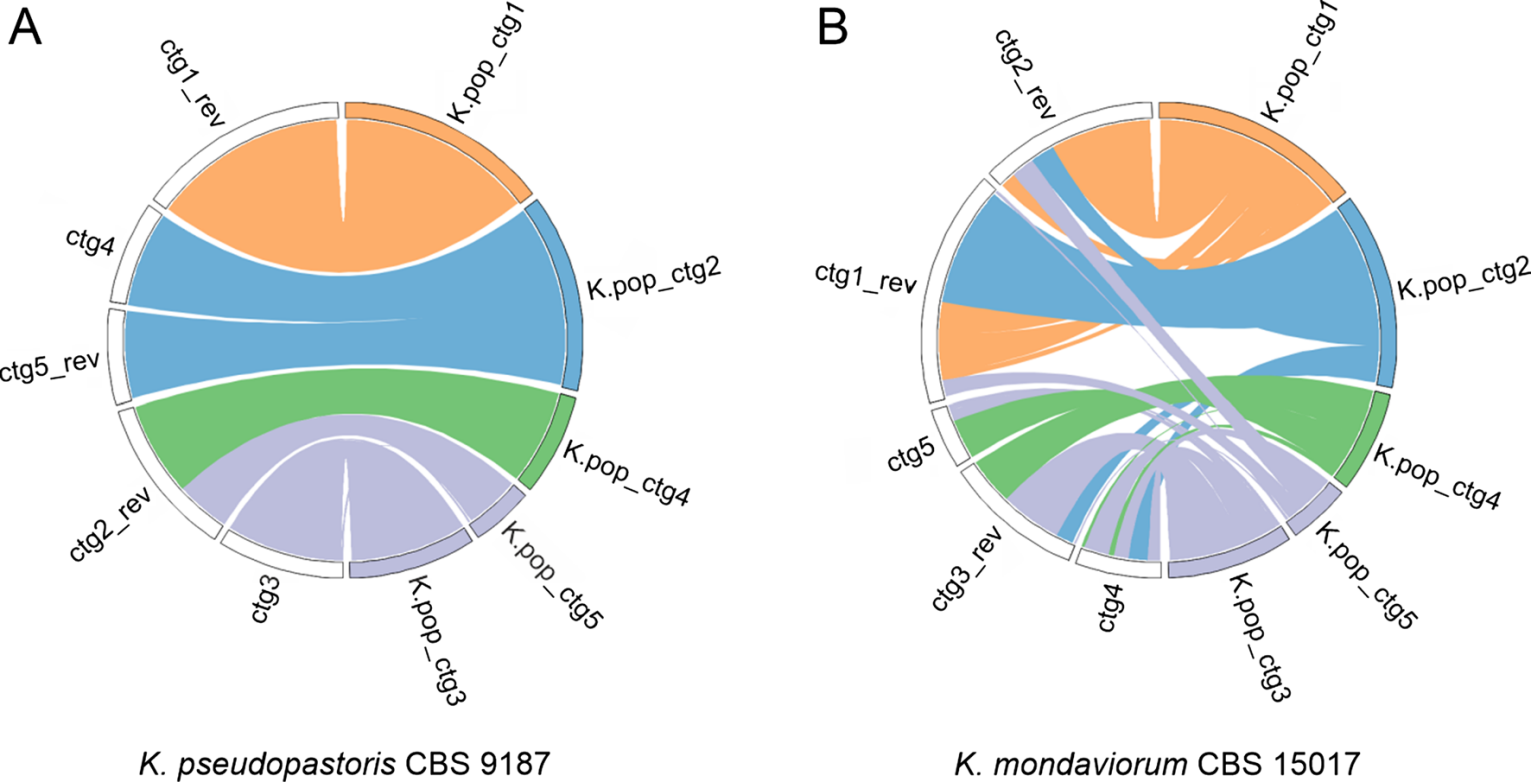
Whole-genome comparison of *K. pseudopastoris* CBS 9187 (**A**) and *K. mondaviorium* CBS 15017 (**B**) against *K. populi* CBS 12362. The order and coloring of *K. populi* contigs were assigned after inspecting the comparisons with *K. phaffii* (remains preliminary due to heavy rearrangements). Contigs of *K. pseudopastoris* and *K. mondaviorum* were ordered and oriented manually to achieve congruence with *K. populi* as far as possible. *ctg* contig, *K. pop* *K. populi*, *rev* reverse

Genome comparisons of the more distant species *K. pseudopastoris*, *K. populi*, and *K. mondaviorum* to *K. phaffii* as reference showed a larger number of structural differences (Fig. 2F–H), and a direct assignment of contigs to *K. phaffii* chromosomes was not possible. However, when comparing *K. pseudopastoris* to *K. populi*, there appeared no major structural difference (Fig. 3A), and *K. mondaviorum* compared to *K. populi* showed fewer rearrangements than compared to *K. phaffii* (Fig. 3B). Although the genomic distance between *K. mondaviorum* and *K. populi* was smaller than the distance between the clusters of *K. phaffii* and *K. pastoris*, there was much more genomic rearrangement between *K. mondaviorum* and *K. populi* than between *K. phaffii* and *K. pastoris*. Each contig of *K. populi* showed similarity to several different contigs of *K. mondaviorum*, e.g., parts of *K. populi* contig2 matched in four different contigs of *K. mondaviorum*. According to the position in the sequence-based tree, the differences between these species were more obvious on the structural level than on the sequence level.

### Sequence divergence between *Komagataella* species

To assess the genomic divergence on the sequence level we used *K. phaffii* CBS 7435 as reference sequence for comparison with each of the eight assembled genomes (Table 3). The divergence based on sequence alignments resulted in three groups in terms of similarity with the closest to the reference being *K. phaffii* UWOPS 03-328y3 and *K. kurtzmanii* CBS12817, the second closest group represented by the *K. pastoris* strains and *K. ulmi* CBS 12361, and the most distant group comprising *K. mondaviorum* CBS 15017, *K. populi* CBS 12362, and *K. pseudopastoris* CBS 9187.

**Table 3:** Average sequence identity in matching regions ≥ 1000 bp between each of the listed genomes and the chromosomes of *K. phaffii* CBS 7435

	Chr1			Chr2			Chr3			Chr4		
	% id	% cov	# m	% id	% cov	# m	% id	% cov	# m	% id	% cov	# m
*K. mondav.* CBS 15017	83.4	71.5	742	83.2	72.0	653	83.5	70.4	600	83.3	67.9	472
*K. ulmi* CBS 12361	90.4	94.5	229	90.2	94.3	213	90.5	97.3	163	90.3	92.6	158
*K. populi* CBS 12362	83.0	67.6	740	82.9	69.1	639	83.0	67.7	594	83.1	64.8	449
*K. kurtzm.* CBS 12817	97.9	88.5	19	97.9	75.4	16	98.0	95.5	24	97.9	90.1	16
*K. phaffii* 03-328y3	99.4	94.0	13	99.4	97.0	11	99.4	72.5	12	99.3	94.8	16
*K. pastoris* DSMZ 70877	90.1	94.3	233	90.0	94.0	248	90.0	94.3	163	90.0	93.0	166
*K. pastoris* CBS 9178	90.1	93.8	228	90.0	94.0	238	90.0	94.0	174	90.0	92.5	168
*K. pseudopastoris* CBS 9187	83.1	69.3	751	83.0	69.9	655	83.2	68.5	591	83.1	65.1	451

*% id* sequence identity in percent, *% cov* covered fraction of the reference chromosome, *# m* number of matching regions

### Phenotypic characterization of 25 *Komagataella* strains

**Fig. 4:**
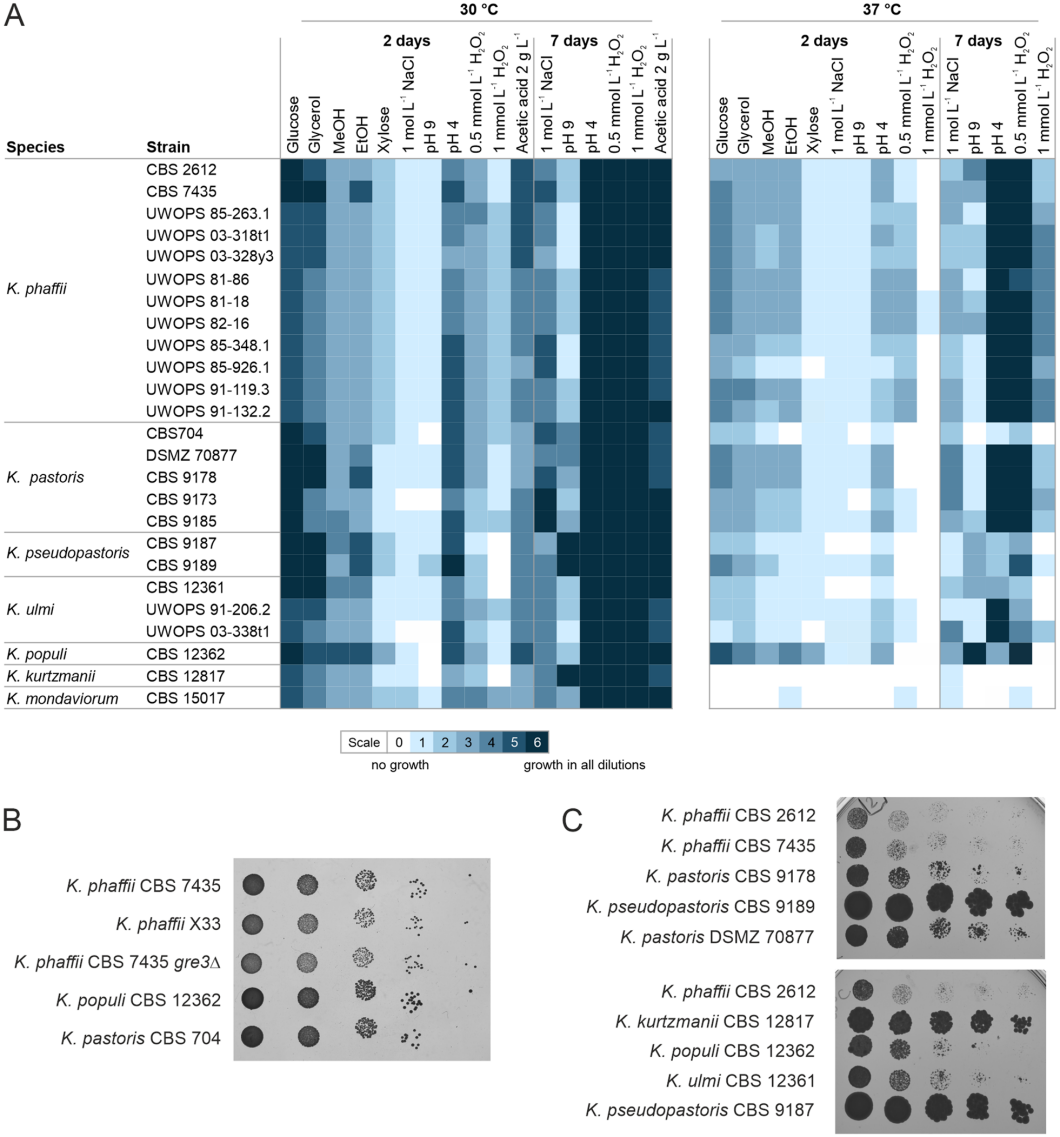
**A** Summary of spotting assays at 30 and 37 °C. Cell growth was evaluated after 2 and 7 days of incubation according to the following criteria: 0 = no growth (white), 1–5 rating depending on the dilution down to which growth was observed, 6 = large colonies in every dilution (dark blue). All plates with inhibitors contained glucose as carbon source. **B** Growth on xylose as the only carbon source. Plate after incubation at 30 °C for 3 days. **C** Growth at pH 9. Plates after incubation at 30 °C for 7 days

For characterization of the phenotypic diversity within the 25 *Komagataella* strains, spotting assays on different carbon sources and in the presence of high salt concentration, oxidative stress, and high and low pH conditions were performed. Colony formation and density were evaluated after 2 and 7 days of incubation at optimal (30 °C) or increased growth temperature (37 °C). Figure 4 shows the results of the growth tests. Overall, a rather homogenous growth behavior was observed under most conditions tested. As expected, at 30 °C all strains could grow well on glucose and glycerol as carbon sources. Growth was slightly reduced on plates with methanol, but to a similar level for all strains except *K. populi* CBS 12362, which showed a better growth on methanol. Growth on ethanol was similar to methanol for most strains. Only *K. phaffii* CBS 7435, *K. pastoris* CBS 9178, the two *K. pseudopastoris* strains, and *K. populi* CBS 12362 showed superior growth compared to the other strains tested. Interestingly, although *Komagataella* species are generally described as non-xylose utilizing yeasts, slow growth on xylose as the sole carbon source was observed for all the strains. However, the growth was much weaker than for the other carbon sources tested. Compared to all other strains tested, growth on xylose was slightly better for *K. pastoris* CBS 704 and *K. populi* CBS 12362 (Fig. 4B).

Cell growth was strongly inhibited by 1 mol L^−1^ NaCl after 2 days of incubation at 30 °C, but most strains were able to adapt to the high salt concentration within 7 days of incubation. Also, the medium with pH 9 had a strong inhibitory effect after 2 days, which both *K. pseudopastoris* strains and *K. kurtzmanii* CBS 12817 could completely overcome until day 7 (Fig. 4C). As expected, all strains showed a high tolerance against acidic pH, where small differences in colony growth could only be observed after 2 days of incubation. Also, all strains were able to grow in the presence of 2 g L^−1^ acetic acid, with *K. populi* CBS 12362 showing the fastest growth after 2 days at 30 °C. Resistance to oxidative stress was tested by incubation on plates with 0.5 and 1 mmol L^−1^ hydrogen peroxide, which had an inhibitory effect after 2 days, which was no longer visible after 7 days, likely due to evaporation of the inhibitor. In this experiment, *K. phaffii* UWOPS 85-263.1 and *K mondaviorum* CBS 15017 stood out as being more tolerant to 0.5 mmol L^−1^ hydrogen peroxide.

Additionally, all spotting assays were also performed at 37 °C (Fig. 4A). As expected, cell growth was generally worse at elevated temperature. When comparing cell growth of the different strains relative to each other, strains showing reduced growth at 30 °C also showed reduced growth at 37 °C on the different carbon sources as well as on the plates with an inhibitory environment. Only *K. populi* CBS 12362 grew to relatively high densities on the different carbon sources. As described previously, *K. kurtzmanii* and *K. mondaviorum* were not able to grow at 37 °C under any of the conditions tested [1, 24]. Similar to 30 °C, nearly every strain, except *K. pastoris* CBS 704, both *K. pseudopastoris* strains, *K. ulmi* CBS 12361 and *K. populi* 12362, was resistant to low pH. Likewise, only *K. pastoris* CBS 704, *K. pseudopastoris* CBS 9187 and the *K. ulmi* strains showed reduced growth in the presence of 0.5 mmol L^−^1 hydrogen peroxide at 37 °C.

### *K. populi* CBS 12362 shows faster growth on xylose than the other 24 strains

**Fig. 5:**
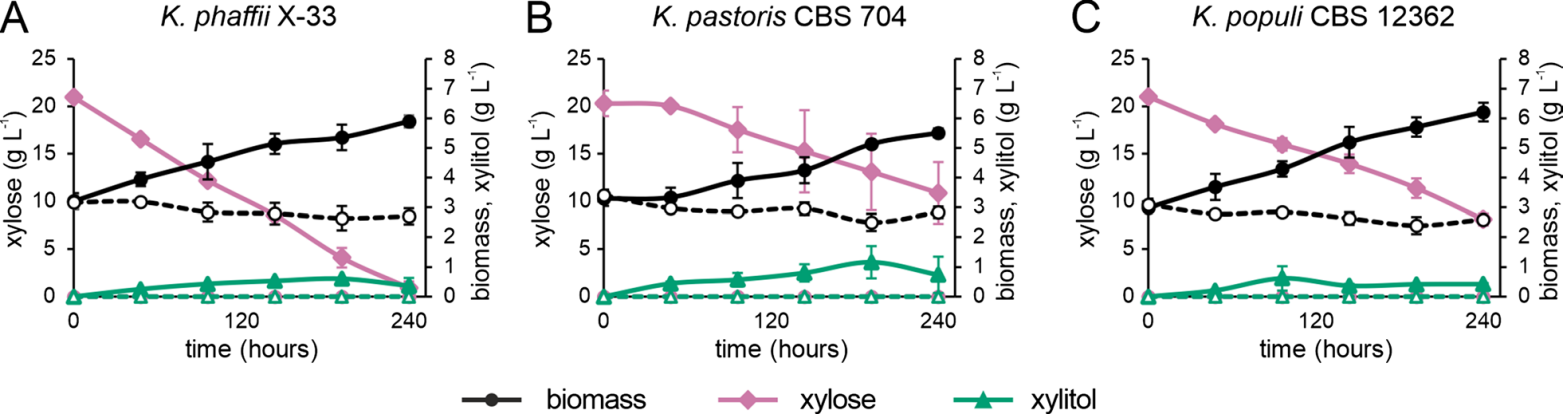
Growth, xylose consumption and xylitol production of *K. phaffii* X-33 (**A**), *K. pastoris* CBS 704 (**B**), and *K. populi* CBS 12362 (**C**) in YNB with 2% xylose (continuous lines with filled symbols). Dashed lines with empty symbols indicate samples from the control cultures in YNB with no addition of carbon source. Data represent the average of three biological replicates with standard deviation

Xylose is a highly abundant substrate for biotechnological processes. Engineering of efficient xylose utilization in *Komagataella phaffii* has previously been achieved by the introduction of the xylose isomerase pathway [11]. To our knowledge, however, growth of non-engineered *K. phaffii* cells solely on xylose was only reported for experiments performed in complex medium [11]. Based on the previous spotting assay results, strains of three different *Komagataella* species were chosen for a more detailed investigation of xylose utilization. Growth of *K. phaffii* X-33 as industrially relevant strain from the *K. phaffii* CBS 7435 background, *K. pastoris* CBS 704 and *K. populi* CBS 12362 was analyzed in minimal medium containing 20 g L^−1^ xylose in shake flasks with an initial OD_600_ of 12 (circa 3.5 g L^−1^ yeast dry mass) (Fig. 5).

No cell growth and metabolite formation were observed in the control conditions without a carbon source. *K. phaffii* X-33 was the best xylose consumer, using up an average of 95.70% ± 3.2 of the available xylose for nearly one doubling (1.85 ± 0.08-fold) within 10 days of incubation. *K. pastoris* CBS 704 had the slowest growth and xylose uptake in comparison to the other two strains (1.64 ± 0.13-fold and 47.1% ± 13.2 of the xylose, respectively). *K. populi* CBS 12362 showed the fastest growth and doubled the number of cells (2.04 ± 0.09-fold), consuming 61.4% ± 3.0 of the available xylose in the process. Xylitol was detected as a by-product with a final average of 0.51 ± 0.16 g L^−1^ in all three strains, with maximum production between 96 and 192 h of cultivation. *K. pastoris* CBS 704 was distinctively the best xylitol producer reaching 1.16 ± 0.54 g L^−1^ after 192 h of cultivation. Other metabolites like ethanol, acetic acid, and glycerol could not be detected as fermentation products.

To identify potential reasons for the observed differences in xylose utilization between *K. phaffii* X-33 and *K. populi* CBS 12362, the sequences of putative xylose pathway genes and their transcript levels were analyzed. Orthologs of xylose reductase, xylitol dehydrogenase, and xylulokinase have previously been identified in *K. phaffii* [11, 20]. We used the protein sequences of xylose reductase (length of 318 amino acids), xylitol dehydrogenase (363 amino acids) and xylulokinase (623 amino acids) from the xylose utilizing yeast *S. stipitis* to identify orthologous sequences in the newly sequences *Komagataella* species. According to sequence homology we inferred the putative xylose reductase gene *GRE3* (PP7435_Chr3-0488, PAS_chr3_0744) encoding a 319 amino acid protein (320 amino acids in *K. ulmi*) in all our *Komagataella* assemblies with around 68% sequence identity to *S. stipitis* xylose reductase (Additional file 1: Table S2, Figure S2A). Interestingly, the deletion of *GRE3* in *K. phaffii* CBS 7435 did not abolish but only reduce cell growth and spot density on xylose plates (Fig. 4B), indicating the presence of at least one other enzyme capable of catalyzing the xylose reductase reaction. Indeed, homology-based search identified the NADPH-dependent aldo–keto reductases Ypr1, PP7435_Chr2-0714 and PP7435_Chr4-0551 as potential candidates, sharing 29–37% sequence identity with *S. stipitis* xylose reductase (Additional file 1: Table S2, Figure S3). Based on sequence homology, *SOR1* (PP7435_Chr1-0597, PAS_chr1-1_0490) was identified as putative xylitol dehydrogenase gene in all *Komagataella* assemblies. It encodes a 348 amino acid protein (409 amino acids in *K. pastoris*) with 54–55% sequence identity to the *S. stipitis* protein (Additional file 1: Table S3, Figure S2B). The inferred xylulokinase orthologs Xks1 (PP7435_Chr1_0598, PAS_chr1-1_0280) have a size of 605, 607 or 617 amino acids in the different *Komagataella* species and share 49–50% sequence identity with the *S. stipitis* protein (Additional file 1: Table S4, Figure S2C). Interestingly, the *SOR1* and *XKS1* genes were found next to each other in all the *Komagataella* assemblies analyzed. The impact of sequence variation between the different *Komagataella* enzymes on xylose utilization remains to be investigated.

Transcript levels of the putative xylose pathway genes in the presence of glucose or xylose as a carbon source were analyzed by quantitative PCR. For this, three clones of each strain were cultivated in YPD and then transferred to minimal medium with 20 g L^−1^ glucose or xylose. Samples for RNA extraction were taken after 6 h in minimal medium.

**Fig. 6:**
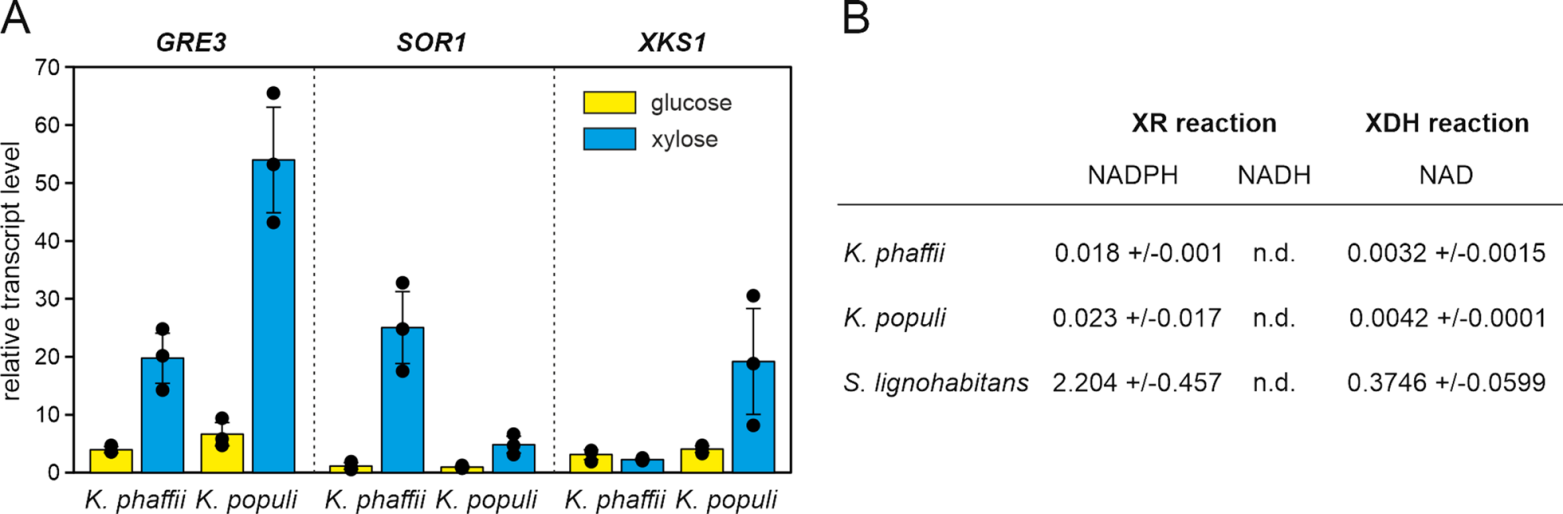
**A** Relative transcript levels of putative xylose pathway genes in *K. phaffii* X-33 and *K. populi* CBS 12362. Transcript levels are given as fold change compared to the average *SOR1* transcript level in *K. phaffii* X-33 grown in minimal medium with glucose. Error bars represent the standard deviation of three biological replicates shown as individual data points. **B** Specific enzyme activities of cell-free extracts prepared from cells grown in minimal medium with xylose. Cultures of the xylose utilizing yeast *S. lignohabitans* grown in the same medium were used as a control. Values are given as U mg^−1^ total protein. Errors represent the standard deviation of three biological replicates. *Nd* activity not detectable

Figure 6A shows the relative transcript levels of the putative xylose pathway genes in *K. phaffii* X-33 and *K. populi* CBS 12362. When comparing the transcript levels of the two different strains in xylose medium, transcript levels of *GRE3* and *XKS1* were higher in *K. populi* CBS 12362 than in *K. phaffii* X-33, while *SOR1* levels were higher in *K. phaffii* X-33. Expression of all genes, except *K. phaffii XKS1*, was upregulated at least 4.8-fold in xylose medium when compared to the expression of the same gene in glucose medium. The absence of induction of *XKS1* expression in *K. phaffii* X-33 could be a reason for the slower growth of this strain on xylose.

Additionally, enzyme activities of xylose reductase and xylitol dehydrogenase were measured in cell-free extracts of cultures grown in YNB with 20 g L^−1^ xylose. Cell-free extracts of the xylose-utilizing yeast *S. lignohabitans* CBS 10342 grown under the same conditions were used as a positive control (Fig. 6B). As expected from the slow growth on xylose, the measured specific activities were very low for both reactions and around 100 times lower than for the positive control *S. lignohabitans*. Also, no significant differences in specific activity between *K. phaffii* X-33 and *K. populi* CBS 12362 could be detected. In many yeasts the reduction of xylose depends on NADPH as the preferred cofactor, however, efficient NADH-dependent xylose conversion has also been described for some species [25, 26]. For *K. phaffii* X-33 and *K. populi* CBS 12362 cell-free extracts, xylose reductase activity could only be determined with NADPH, indicating that the activity with NADH is either much lower or absent in these strains.

### Xylose assimilation in ***K. phaffii*** X-33—^13^C labeling

To show the incorporation of carbon from xylose into cellular metabolites and yeast biomass, an isotope labeling experiment with 1-^13^C xylose was performed. After a preculture in YP medium with glycerol followed by an overnight culture in xylose medium for adaption, *K. phaffii* X-33 cultures were grown in triplicates in minimal medium with ^12^C or 1-^13^C xylose as carbon source for 10 days before sampling and quenching of biomass for metabolite analysis. The total ^13^C content of the biomass was measured by Elemental Analysis—Isotope Ratio Mass Spectrometry (EA-IRMS). For the samples grown in medium with 1-^13^C xylose, an average total ^13^C content of 9% was measured, which is slightly higher than the expected value of approximately 5% calculated from the biomass increase during the cultivation. As expected, the total ^13^C content of the samples grown with ^12^C xylose was around 1.1%, which represents the exact natural ^13^C abundance [27].

**Fig. 7:**
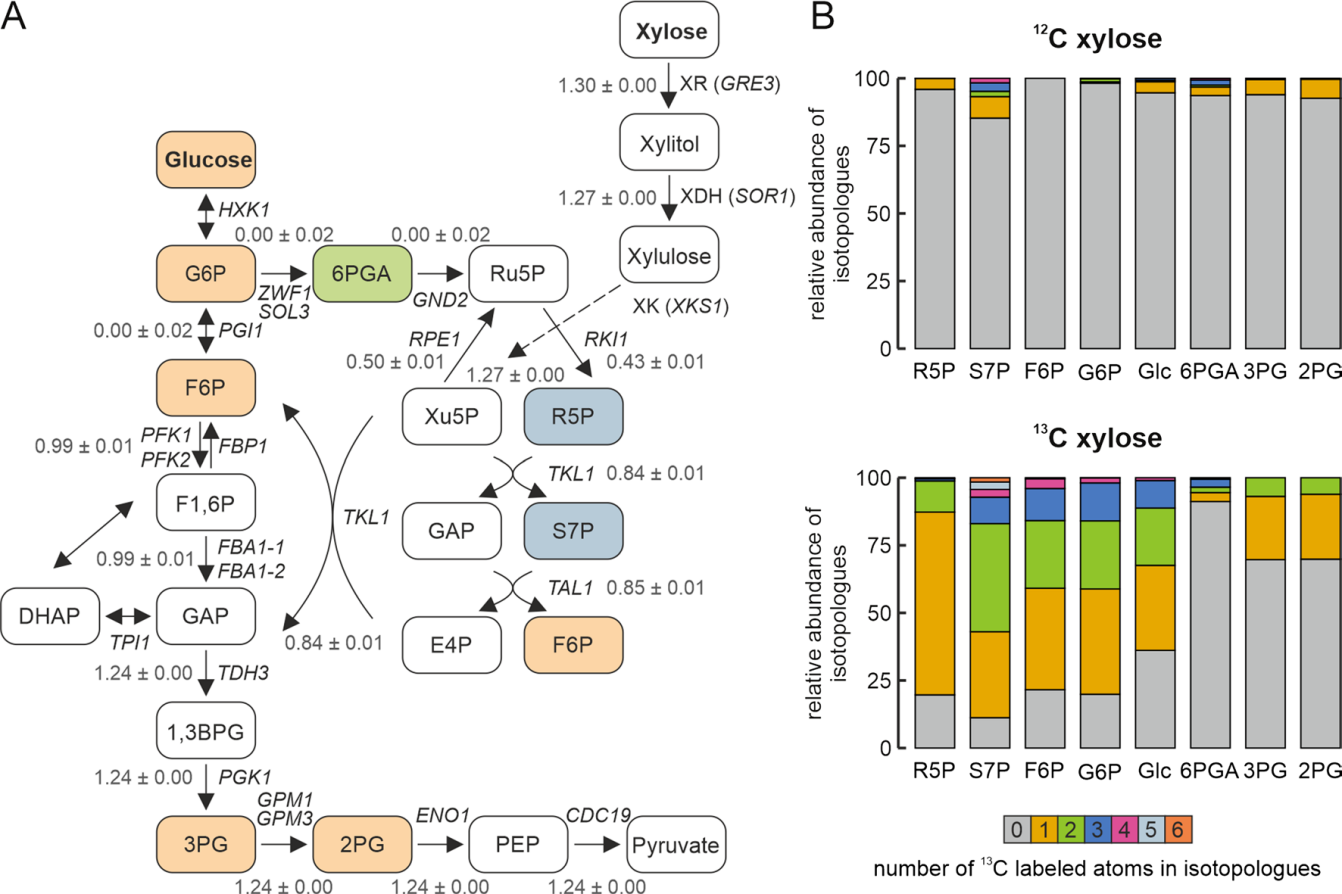
Xylose assimilation in *K. phaffii* X-33—^13^C labeling and modeling of metabolic fluxes. **A** Putative xylose utilization pathway. The isotopologue distribution of all colored metabolites was measured by mass spectrometry. The colors indicate the pathway to which the metabolites mainly belong (orange—glycolysis, green—oxidative pentose phosphate pathway, blue—non-oxidative pentose phosphate pathway). Absolute specific flux rates per carbon atom (mmol g^−1^ h ^−1^) with 95% confidence intervals were calculated by ^13^C metabolic flux analysis. Values of each reaction refer to the total number of carbon atoms involved in the corresponding reaction. For reversible fluxes, only net fluxes are shown. **B** Relative abundance of ^13^C labeled isotopologues in selected metabolites after cultivation on ^12^C or 1-^13^C D-xylose

The incorporation of ^13^C from xylose into the metabolites of the pentose phosphate pathway and glycolysis was confirmed by gas chromatography (GC)—time of flight mass spectrometry (TOFMS) mass spectrometry isotopologue distribution analysis. An overview of the core metabolism including the putative xylose assimilation reactions is shown in Fig. 7A. The labeling pattern of relevant metabolites confirmed the incorporation of ^13^C from xylose into intermediates of the non-oxidative pentose phosphate pathway (Fig. 7B, Additional file 1: Table S5) as well as glucose, glucose-6-phosphate, 3-phosphoglycerate, and 2-phosphoglycerate as intermediates of glycolysis. Hardly any ^13^C label could be detected in 6-phosphoglycerate, showing that the carbon is entering the pentose phosphate pathway at the level of pentoses, while the oxidative part of the pathway is less active when cells are grown on xylose. As expected, a very low ^13^C content was measured for metabolites from control cultures grown on standard ^12^C xylose. For comparison, metabolic fluxes were also calculated using a previously published stoichiometric model of the central carbon metabolism with the addition of the xylose utilization reactions (Fig. 7A, Additional file 2) [28]. The prediction showed no metabolic flux through the oxidative pentose phosphate pathway, which corresponds to the results obtained from the 1-^13^C labeling experiment, where very little ^13^C was detected in 6-phosphoglycerate. A deviation from the model was only observed for glucose-6-phosphate and glucose, where the label was clearly detected in the metabolite analysis.

## Discussion

This study provides an overview of the genetic and phenotypic diversity of *Komagataella* strains of all seven described species available from public strain collections. Genome sequencing of eight different strains by PacBio whole genome sequencing resulted in high-quality genome assemblies, including the first published whole genome sequences of strains belonging to *K. kurtzmanii*, *K. mondaviorum*, and *K. ulmi.* Phylogenetic analysis based on a representative set of subsequences overall confirmed the phylogenetic relationship between the species as determined by marker gene sequences [1]. However, the species *K. mondaviorum*, *K. populi*, and *K. pseudopastoris* showed a larger distance to the subtrees of *K. phaffii* and *K. pastoris* than in the analysis based on marker genes. The newly sequenced *K. phaffii* and *K. kurtzmanii*, and *K. pastoris* and *K. ulmi* strains, respectively, share the global differences in genome structure previously described for *K. phaffii* and *K. pastoris* [5]. The small number of chromosomal rearrangements between these four species allowed the assignment of chromosome number and orientation based on the published reference genomes. While the number of rearrangements in the other three species was too high for chromosome assignment based on the *K. phaffii* reference genome, pairwise comparison of the genome structure showed no large rearrangements between *K. pseudopastoris* and *K. populi*, while the extent of rearrangement between *K. populi* and *K. mondaviorum* was found to be higher than between *K. phaffii* and *K. pastoris*. Overall, the phylogenetic relationship of the species as shown in Fig. 1 is consistent over the different types of genome comparisons performed within this study.

The phenotypic diversity within the genus was analyzed by growth assays on different carbon sources and different stress conditions. It has previously been suggested that different species of *Komagataella* cannot be distinguished based on growth tests alone [1]. Accordingly, the observed growth was very similar for most of the conditions tested, especially for common carbon sources like glucose, glycerol, and methanol. Only on ethanol plates, some strains stood out as better growers at 30 °C. Clear differences could also be observed in tolerance to high pH and oxidative stress conditions. For all the strains growth was reduced at 37 °C, but only *K. kurtzmanii* CBS 12817 and *K. mondaviorum* CBS 15017 were unable to grow at this temperature in any of the conditions tested, as previously described for growth on glucose [1, 24]. *K. populi* CBS 12362 stood out as one of the fastest growing strains on all carbon sources and in most stress conditions tested. It will be interesting to investigate if these phenotypic characteristics are strain or species specific as soon as there are more isolates available.

The most intriguing observation from the growth assays was the slow but significant cell growth on minimal medium with xylose as the sole carbon source. Generally, *Komagataella* species are not known to utilize xylose and so far, growth on xylose has only been characterized for one *K. phaffii* strain in complex medium or after the introduction of a heterologous xylose isomerase pathway [11]. Detailed analysis of cell growth in liquid minimal medium showed long doubling times of around 10 days. Within this time, all three strains consumed xylose, with *K. phaffii* X-33 consuming more than 95% of the xylose in the medium while *K. populi* CBS12362 showed slightly faster growth with lower xylose consumption. The formation of xylitol as a byproduct was similar in all three strains and indicated the assimilation of xylose through an oxidoreductase pathway as described for other xylose consuming yeasts like *S. stipitis* [13, 29]. Using homology-based search, orthologs of xylose reductase, xylitol dehydrogenase and xylulokinase could be identified in all *Komagataella* species. Interestingly, many yeast species not known to utilize xylose have one or more copies of xylose pathway genes in their genomes, indicating that efficient utilization of xylose also depends on other factors like gene regulation and enzyme activity [20]. In *K. phaffii* X-33 and *K. populi CBS* 12362, the expression of putative xylose pathway genes, except for *K. phaffii* X-33 *XKS1*, was upregulated in xylose medium. Also, xylose reductase and xylitol dehydrogenase activities were measurable in cell free extracts, with activities around 100 times lower than in the xylose-utilizing yeast *S. lignohabitans* [30]. Upregulation of *GRE3* and *XKS1* gene expression and enzyme activity in the presence of xylose were both slightly higher in *K. populi* CBS 12362, which might explain the faster growth. Labeling experiments and metabolite analysis with ^13^C xylose showed high levels of ^13^C incorporation into metabolites of the non-oxidative pentose phosphate pathway and glycolysis, confirming the utilization of xylose via the oxidoreductase pathway. Further experiments are required to clearly determine the regulation of xylose utilization in *Komagataella*. Also, it remains to be tested if the overexpression of the native xylose utilization pathway genes can improve the growth to allow production of recombinant proteins or metabolites with xylose as carbon source.

Overall, our data on the phenotypic and genotypic diversity will provide the basis for use of so-far neglected *Komagataella* isolates with interesting characteristics and elucidation of the genetic determinants of improved growth and stress tolerance for targeted strain improvement.

## Materials and methods

### Yeast strains

All *Komagataella* isolates used in this study are listed in Table 4. All strains are available from public culture collections and were obtained from the Westerdijk Fungal Biodiversity Institute (CBS-KNAW, Netherlands), the German Collection of Microorganisms and Cell Cultures (DSMZ, Germany), and the University of Western Ontario, Department of Biology Yeast Culture Collection (UWOPS, Canada). The *K. phaffii* CBS 7435 *gre3*Δ strain was generated using a Golden*Pi*CS CRISPR/Cas9 vector with a gRNA targeting *GRE3* (PP7435_Chr3-0448) and a homology template for complete removal of the coding sequence [31, 32]. The deletion of *GRE3* was confirmed by PCR.

**Table 4:** List of strains used in this study

Strain name		Isolated from	Origin	Source
***K. phaffii***	**CBS 2612**	*Quercus kelloggii*	California, USA	CBS-KNAW
*K. phaffii*	CBS 7435	Derivative of CBS 2612	USA	NRRL
*K. phaffii*	UWOPS 85-263.1	*Quercus emoryi*	Arizona, USA	UWOPS
*K. phaffii*	UWOPS 03-318t1	*Quercus rubra*	Ontario, Canada	UWOPS
*K. phaffii*	UWOPS 03-328y3^b^	*Quercus rubra*	Ontario, Canada	UWOPS
*K. phaffii*	UWOPS 81-86	*Quercus rubra*	unknown	UWOPS
*K. phaffii*	UWOPS 81-18	*Quercus rubra*	Ontario, Canada	UWOPS
*K. phaffii*	UWOPS 82-16	*Quercus rubra*	Ontario, Canada	UWOPS
*K. phaffii*	UWOPS 85-348.1	*Quercus emoryi*	Arizona, USA	UWOPS
*K. phaffii*	UWOPS 85-926.1	*Quercus emoryi*	Arizona, USA	UWOPS
*K. phaffii*	UWOPS 91-119.3	*Quercus rubra*	Ontario, Canada	UWOPS
*K. phaffii*	UWOPS 91-132.2	*Quercus rubra*	Ontario, Canada	UWOPS
***K. pastoris***	**DSM 70382 (CBS 704)**	*Aesculus *species^a^	France	DSMZ
*K. pastoris*	DSM 70877^b^	*Fagus *species	Germany	DSMZ
*K. pastoris*	CBS 9178^b^	*Quercus *species	Hungary	CSB-KNAW
*K. pastoris*	CBS 9173	*Acer platanoides*	Hungary	CSB-KNAW
*K. pastoris*	CBS 9185	*Fagus sylvatica*	Hungary	CSB-KNAW
***K. pseudopastoris***	**CBS 9187**^b^	*Salix alba*	Hungary	CSB-KNAW
*K. pseudopastoris*	CBS 9189	*Salix alba*	Hungary	CSB-KNAW
***K. ulmi***	**CBS12361**^b^	*Ulmus americana*	Illinois, USA	CSB-KNAW
*K. ulmi*	UWOPS 91-206.2	*Quercus rubra*	Ontario, Canada	UWOPS
*K. ulmi*	UWOPS 03-338t1	*Quercus rubra*	Ontario, Canada	UWOPS
***K. populi***	**CBS 12362**^b^	*Populus deltoides*	Illinois, USA	CSB-KNAW
***K. kurtzmanii***	**CBS 12817**^b^	Fir flux	Arizona, USA	CSB-KNAW
***K. mondaviorum***	**CBS 15017**^b^	*Populus deltoides*	California, USA	CSB-KNAW
*S. lignohabitans*	CBS 10342	Decayed log	Illinois, USA	CSB-KNAW

Species type strains are shown in bold

*CBS-KNAW* Westerdijk Fungal Biodiversity Institute Collection, *NRRL* Agricultural Research Service Culture Collection, *UWOPS* University of Western Ontario, Department of Biology Yeast Culture Collection, *DSMZ* German Collection of Microorganisms and Cell Cultures

^a^As described in the original publication [33]. Often reported as *Castanea* species, probably due to mistranslation from the original French description

^b^Newly sequenced strains

### Genome sequencing

The strains selected for whole-genome sequencing are indicated in Table 4. Cells were grown in YPD medium (yeast extract 1%, peptone 2%, glucose 2%) overnight and genomic DNA was extracted using the Genomic-tip 100/G kit (Qiagen) according to the manufacturer’s protocol. Sequencing libraries were prepared with the SMRTbell Express Template Prep Kit 2.0 together with the Barcoded Overhang Adapter Kits 8A/8B (PacBio) and sequenced on a PacBio Sequel RSII instrument. The genomes were assembled with the HGAP4-pipeline included in PacBio SMRTLink software v8.0 using standard parameters [34]. Library preparation, sequencing, and genome assembly were performed by the Next Generation Sequencing Facility at Vienna BioCenter Core Facilities (VBCF), a member of the Vienna BioCenter (VBC), Austria. Sequencing reads and genome assemblies are available from NCBI database under the BioProject accession PRJNA770850. Mitochondrial sequences as identified through the NCBI Contamination Screen were removed from the assemblies.

### Comparative genome analysis

As reference genomes, we used the assemblies of *K. phaffii* CBS 7435 (NCBI identifier ASM170808v1) and *K. pastoris* CBS 704 (ASM170810v1). Genome comparisons were performed using minimap2 v2.12-r827 [35] with default parameters. Matches with mapping quality > 0 and spanning ≥ 1 kbp were kept for further analysis. Circular plots were generated using Circos v0.69-8 [36] based on the minimap2 comparisons whereby only matches ≥ 10 kbp were displayed. Contigs without a match were removed. To assess the sequence divergence, the eight assembled genomes were matched against *K. phaffii* CBS 7435 as reference using nucmer v3.1 with parameter -g 1000, delta-filter with parameters -l 100 -o 0 -q, and show-coords with parameters -qldcoHT from the mummer suite [37]. Only matches of length 1 kbp or larger were evaluated. For calculation of % sequence identity per chromosome the sequence identity in each match region was multiplied by the match length, and the sum for all match regions within one chromosome was divided by the total match length per chromosome. Orthologous *Komagataella* genes of XP_001385181.1 (XR), XP_001386982.1 (XDH), and XP_001387325.2 (XKS) from *Scheffersomyces stipitis* were identified by searching the eight assemblies using tblastn v2.12.0 [38] followed by manual selection of start- and stop-codons. The coding sequences of the identified genes were translated using standard genetic code and used as input for multiple sequence alignments with Clustal Omega [39].

The genome of *Citeromyces matritensis* NRRL Y-2407 (NCBI id ASM370516v1) was used as outgroup in the phylogenetic analysis. Pairwise distances were determined between all genome assemblies employing the *dist* function implemented in Mash [22]. The distance-based phylogenetic tree was calculated using the Fitch algorithm [40] included in PHYLIP [41] with ten times jumbling (jumble seed 23893) and global rearrangements. For tree display, we used the Newick utilities toolkit [42]. Custom scripting and analyses were performed using Perl 5 and bash. The read length distribution (Additional file 1: Figure S1) was plotted with R v3.6.0 [43].

### Spotting assays

For spotting assays, fresh colonies from glycerol stocks were resuspended in PBS or grown in YPD medium (yeast extract 1%, peptone 2%, glucose 2%) overnight. Cells were washed twice and diluted in PBS to OD_600_ 0.3 in a 96-well plate. Using a multichannel pipette, five series of 1:10 dilutions were done (up to 1:10^5^). Starting from the highest dilution, 4 µL of each dilution were pipetted onto YNB agar plates (YNB without amino acids and ammonium sulfate 3.4 g L^−1^, ammonium sulfate 10 g L^−1^, potassium phosphate buffer 0.1 mol L^−1^ pH 6, biotin 0.4 mg L^−1^, agar–agar 2%) supplemented with different carbon sources and/or inhibitors. To test growth on different carbon sources, plates were supplemented with 2% glucose, glycerol, methanol, or xylose. For inhibitor tests, YNB agar with 2% glucose was used. The final concentrations of inhibitors were: NaCl 1 mol L^−1^, H_2_O_2_ 0.5 and 1 mmol L^−1^, acetic acid 2 g L^−1^. Plates were incubated at 30 or 37 °C for 168 h. Pictures were taken every 24 h on a scanner EPSON^®^ perfection V750 PRO.

### Growth assays in shake flask

Fresh colonies from glycerol stocks were used to inoculate 50 mL YPG (yeast extract 1%, peptone 2%, glycerol 2%) and incubated at 25 °C and 180 rpm overnight. Cells from the glycerol pre-cultures were washed twice and used to inoculate 25 mL of YNB medium (YNB without amino acids and ammonium sulfate 3.4 g L^−1^, ammonium sulfate 10 g L^−1^, potassium phosphate buffer 0.1 mol L^−1^ pH 6, biotin 0.4 mg L^−1^) supplemented with 2% xylose or no carbon source at a starting OD_600_ of 12. Cultures were incubated at 25 °C and 180 rpm for 10 days (240 h). A mixture of 50 U mL^−1^ penicillin and 50 µg mL^−1^ streptomycin (Gibco) were added to the medium to prevent contamination. Cell growth was monitored by measuring OD_600_ and the cell dry mass was determined from the pre-culture and at the end of the cultivation. Xylose consumption and metabolite profiles were monitored by HPLC. For the ^13^C labeling experiment, cells from pre-cultures in YPG were incubated in 30 mL YNB with 2% xylose overnight and then used to inoculate 10 mL YNB with 2% ^12^C or 1-^13^C D-xylose (Sigma-Aldrich) at a starting OD_600_ of 9. Cells were grown at 25 °C and 180 rpm for ten days and samples for OD_600_, cell dry mass, and HPLC analysis were taken at the beginning and end of the cultivation. All growth assays were performed in triplicates.

### Transcript level analysis

For transcript level analysis, three clones per strain were cultivated in YPD medium (yeast extract 1%, peptone 2%, glucose 2%) in shake flasks overnight. Cells were washed and used to inoculate YNB medium (YNB without amino acids and ammonium sulfate 3.4 g L^−1^, ammonium sulfate 10 g L^−1^, potassium phosphate buffer 0.1 mol L^−1^ pH 6, biotin 0.4 mg L^−1^) with 2% glucose or xylose as carbon source. After 6 h of cultivation at 25 °C, cells were harvested and RNA was extracted according to the TRI reagent (Sigma-Aldrich) protocol, followed by DNase treatment with the Ambion DNA-free kit (Invitrogen) and cDNA synthesis with oligo(dT)23 primers (NEB) and the Biozym cDNA synthesis kit. RNA integrity was analyzed by agarose gel electrophoresis. Quantitative PCR was performed on a Rotor-Gene Q instrument (Qiagen) using the Blue S´Green qPCR kit (Biozym). Transcript levels were normalized to *ACT1* expression and relative expression levels were calculated using the average expression of *SOR1* in *K. phaffii* X-33 grown in YNB glucose as reference.

### Enzyme activity assays

Fresh colonies grown in YPD (yeast extract 1%, peptone 2%, glucose 2%) overnight were harvested, washed, and used to inoculate YNB media (YNB without amino acids and ammonium sulfate 3.4 g L^−1^, ammonium sulfate 10 g L^−1^, potassium phosphate buffer 0.1 mol L^−1^ pH 6, biotin 0.4 mg L^−1^) supplemented with 2% xylose at a starting OD_600_ of 10. After 24 h of incubation at 25 °C, cells were harvested and stored at − 20 °C. For protein extraction, approximately 10^8^ cells per sample were washed with PBS and suspended in cell lysis buffer (HEPES 20 mmol L^−1^, NaCl 420 mmol L^−1^, MgCl_2_*6H_2_O 1.5 mmol L^−1^, pH 8) with protease inhibitor (SIGMA*FAST* protease inhibitor cocktail tablets, Sigma-Aldrich). Cell lysis was performed by mechanical cell disruption using glass beads. Cell lysates were centrifuged (19,000*g*, 4 °C, 30 min) and the supernatants were stored on ice. The total protein content of the cell-free extracts was determined using a standard Bradford assay with BSA as a standard (Quick Start Bradford 1 × Dye Reagent, BioRad).

The enzyme activity assays were performed at room temperature in a total volume of 250 µL in 96-well plates as described previously [44]. For xylose reductase activity, the reaction mixture contained 20 µL cell-free extract in triethanolamine buffer (triethanolamine 100 mmol L^−1^, pH 7), 0.2 mmol L^−1^ NADH or NADPH, and 350 mmol L^−1^ xylose. For the xylitol dehydrogenase reaction, 0.5 mmol L^−1^ NAD^+^ and 300 mmol L^−1^ xylitol were used. Fifteen min after the addition of the cofactor, the reactions were started by the addition of the substrate and monitored by measuring the change in absorption at 340 nm. Specific activities were calculated in units per mg of total protein (U mg^−1^). One enzyme unit was defined as the amount of enzyme required to oxidize or reduce 1 μmol of cofactor per minute.

### ^13^C content of biomass

For ^13^C content determination in the yeast biomass, samples were taken at the end of the cultivation and washed twice with HCl 0.1 mol L^−1^ and ultrapure water. Cell pellets of approximately 10 mg of dry biomass were stored at – 20 °C until analysis. The ^13^C/^12^C content of the yeast biomass was analyzed by Imprint Analytics GmbH (Austria) by elemental analysis with isotope ratio mass spectrometry (EA-IRMS).

### GC-TOFMS isotopologue distribution analysis of intracellular metabolites

Sampling and quenching of biomass samples for metabolome analysis were performed as described previously [45]. For the determination of the ^13^C labeling patterns of the metabolites under investigation the method of Mairinger et al. [46] was developed further. Briefly, aliquots of 180 µL (corresponding to 1.7 µg CDW) were dried in 1.5 mL chromatography vials after the addition of 20 µL of a solution of ethoxyamine hydrochloride in pyridine (*c* = 20 mg mL^−1^ pyridine) for protection of the keto and aldehyde carbonyl groups. The dried samples were placed on a cooled rack (7 °C) of the Gerstel MPS2 GC-(Q)TOFMS derivatization robot and autosampler equipped with trays, syringes, and heated agitators for automated two-step derivatization and just in time online injection. Derivatization was performed as follows: ethoximation using 18 µL of O-ethylhydroxylamine hydrochloride in water free pyridine (*c* = 19 mg mL^−1^) (Sigma Aldrich) at 40 °C for 90 min, followed by trimethylsilylation using 42 µL of N-methyl-N-(trimethylsilyl) trifluoroacetamide + 1% trimethylchlorosilane (Thermo Scientific) at 40 °C for 50 min. One µL aliquots of the derivatized samples were injected into an Agilent split/splitless injector with an Ultra Inert straight splitless liner in splitless mode (280 °C, septum purge flow 3 mL min^−1^). The following column setup was used for gas chromatographic separation on an Agilent 7890B GC (Agilent Technologies Inc.): (1) a non-polar deactivated pre-column (3 m × 0.25 µm i.d., Phenomenex), followed by a backflush unit (purged ultimate union, Agilent Technologies Inc.) for connection with (2), the analytical column (Optima^®^ 1 MS Accent (100% dimethylpolysiloxane, 60 m × 250 μm × 0.25 μm, Macherey Nagel), which was connected via a second purged ultimate union T-piece to (3), a non-polar deactivated restrictor column (3 m × 0.18 µm i.d., Phenomenex). Helium 5.0 served as carrier gas and a constant flow of 1.1 mL min^−1^ for the pre-column, 1.2 mL min^−1^ for the analytical column and 1.4 mL min^−1^ for the post column was used. In contrast to Mairinger et al. [46] the following GC-temperature program with a lower initial ramp was employed (total run time 33.2 min): 70 °C for 1 min, 15 °C min^−1^–190 °C, 5 °C min^−1^–225 °C, 3 °C min^−1^–260 °C, 20 °C min^−1^–310 °C, hold for 3 min. The transfer line temperature was set to 280 °C.

An Agilent Technologies 7200B Q-TOF mass spectrometer (Agilent Technologies Inc.) was operated in positive chemical ionization mode using methane as reagent gas employing the settings described previously [47]. Retention times of the metabolites under investigation, mass/charge ratios evaluated for ^13^C isotopologue distribution analysis, and the respective extraction windows are listed in Additional file 1: Table S6.

Peak areas were integrated in Mass Hunter Workstation, Quantitative Analysis (Version 10.1, Agilent Technologies Inc.). In order to obtain the isotopologue distribution of the carbon backbone, integrated peak areas were then corrected for naturally distributed heavy stable isotopes. For that, results were processed using the ICT correction toolbox [48], which corrects for H, N, O, Si, and S isotopes of the derivatized molecule as well as the C isotopes stemming from derivatization (i.e., all C atoms apart from backbone C atoms). Quality control was performed using a cell extract of *K. phaffii* fed with 50:50 ^12^CH_3_OH: ^13^CH_3_OH (Isotopic Solution, Austria), leading to a carbon isotopologue distribution following the Pascal’s Triangle [49]. The relative abundance RA_i_ of each isotopologue i was calculated based on the ICT corrected Peas Areas A_i_ according to Eq. 1 (n = number of carbon atoms in metabolite backbone).

1 $${\mathrm{RA}}_{\mathrm{i}} \left[\mathrm{\%}\right]=\frac{{\mathrm{A}}_{\mathrm{i}}}{{\sum }_{\mathrm{i}=0}^{\mathrm{n}}{\mathrm{A}}_{\mathrm{i}}}$$

### Modeling of metabolic fluxes

The stoichiometric model (Additional file 2) used for the calculation of intracellular fluxes was based on a previously published model of *K. phaffii* central carbon metabolism [28]. For xylose utilization via the oxidoreductase pathway and transport of xylose and xylitol 5 reactions were added (Additional file 2, R25, R48-50, R59). OpenFLUX with standard settings was used for flux calculation and a Monte Carlo algorithm was applied for sensitivity analysis to calculate the confidence intervals (95%) [50]. Average values from GC-TOFMS isotopologue distribution analysis with corresponding standard deviations were used as input data for the flux calculations. All calculations were done with MATLAB (R2019ba, The MathWorks, Inc., Natick, Massachusetts, USA).

## Supplementary Information

**Additional file 1: Figure S1.** Length distribution of PacBio subreads. **Figure S2.** Multiple sequence alignments of xylose pathway enzymes. **Figure S3.** Multiple sequence alignments of the *K. phaffii* CBS 7435 Gre3 homologs. **Table S1.** Pairwise distances between genomes of different *Komagataella* species. **Table S2.** Orthologs of *S. stipitis* xylose reductase (XR) in different *Komagataella* species. **Table S3.** Orthologs of *S. stipitis* xylitol dehydrogenase (XDH) in different *Komagataella* species. **Table S4.** Orthologs of *S. stipitis* xylulokinase (XKS) in different *Komagataella* species. **Table S5.** Relative abundance of isotopologues of selected metabolites after cultivation on ^12^C or 1-^13^C D-xylose. **Table S6.** List of metabolites and corresponding GC retention times (RT), chemical ionization adducts/fragments evaluated for ^13^C isotopologue distribution analysis.

**Additional file 2: **Stoichiometric model of the central carbon metabolism plus xylose utilization reactions.

## Data Availability

The datasets supporting the conclusions of this article are included within the article and its additional files. Whole genome sequencing reads and genome assemblies are available from NCBI database under BioProject accession PRJNA770850.
