# Supplementary material for: Genotypic and phenotypic diversity among Komagataella species reveals a hidden pathway for xylose utilization
Source: Microb Cell Fact. 2022 Apr 25;21:70. doi: 10.1186/s12934-022-01796-3 (PMC9036795; doi:10.1186/s12934-022-01796-3)
Supplement: Supplementary file 1 — Additional file 1: Figure S1. Length distribution of PacBio subreads. Figure S2. Multiple sequence alignments of xylose pathway enzymes. Figure S3. Multiple sequence alignments of the K. phaffii CBS 7435 Gre3 homologs. Table S1. Pairwise distances between genomes of different Komagataella species. Table S2. Orthologs of S. stipitis xylose reductase (XR) in different Komagataella species. Table S3. Orthologs of S. stipitis xylitol dehydrogenase (XDH) in different Komagataella species. Table S4. Orthologs of S. stipitis xylulokinase (XKS) in different Komagataella species. Table S5. Relative abundance of isotopologues of selected metabolites after cultivation on 12C or 1-13C D-xylose. Table S6. List of metabolites and corresponding GC retention times (RT), chemical ionization adducts/fragments evaluated for 13C isotopologue distribution analysis. [file 12934_2022_1796_MOESM1_ESM.pdf]

Supplementary material

Genotypic and phenotypic diversity among *Komagataella* species reveals a hidden pathway for xylose utilization

Lina Heistinger[1,#,+,*], Juliane C. Dohm[2*], Barbara G. Paes[1,3*], Daniel Koizar[1], Christina Troyer[4], Özge Ata[1,5], Teresa Steininger-Mairinger[4], Diethard Mattanovich[1,5]

[1] University of Natural Resources and Life Sciences Vienna (BOKU), Department of Biotechnology, Institute of Microbiology and Microbial Biotechnology, 1190 Vienna, Austria

[2] University of Natural Resources and Life Sciences Vienna (BOKU), Department of Biotechnology, Institute of Computational Biology, 1190 Vienna, Austria

[3] University of Brasilia (UnB), Institute of Biological Sciences, Department of Cell Biology, Brasilia, Brazil

[4] University of Natural Resources and Life Sciences Vienna (BOKU), Department of Chemistry, Institute of Analytical Chemistry, 1190 Vienna, Austria

[5] Austrian Centre of Industrial Biotechnology (acib GmbH), 1190 Vienna, Austria

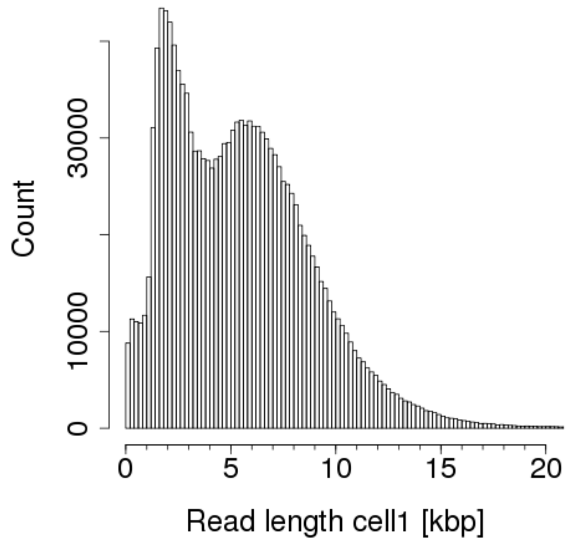

Figure S1: Length distribution of PacBio subreads from one SMRT cell. The other two cells showed similar length profiles.

Table S1: Pairwise distances between genomes of eleven species as determined by Mash dist [1].

	C. *matrit.*	*K. mondav*.	*K. ulmi*	*K. populi*	*K. kurtzm*.	*K. phaffii* (2)	*K. pastoris* (2)	*K. pastoris* (3)	*K. pseudopast*.	Ref_*K.pastoris*	Ref_*K. phaffii*
C. matritensis	0	0.295981	0.263022	0.263022	0.263022	0.263022	0.263022	0.263022	0.263022	0.263022	0.263022
K .mondav.	0.295981	0	0.149777	0.0554283	0.149777	0.145727	0.14383	0.145727	0.052714	0.145727	0.151946
K. ulmi	0.263022	0.149777	0	0.174408	0.0774628	0.0782587	0.0374354	0.0374354	0.167688	0.0374354	0.0786628
K. populi	0.263022	0.0554283	0.174408	0	0.159146	0.151946	0.174408	0.174408	0.0259767	0.182269	0.161822
K. kurtzm.	0.263022	0.149777	0.0774628	0.159146	0	0.0181917	0.0747985	0.0751681	0.159146	0.0751681	0.0182524
K. phaffii (2)	0.263022	0.145727	0.0782587	0.151946	0.0181917	0	0.0759181	0.0762985	0.156619	0.0770708	0.00420918
K. pastoris (2)	0.263022	0.14383	0.0374354	0.174408	0.0747985	0.0759181	0	0.00238081	0.159146	0.0025468	0.0774628
K. pastoris (3)	0.263022	0.145727	0.0374354	0.174408	0.0751681	0.0762985	0.00238081	0	0.159146	0.00274254	0.0778587
K. pseudopast.	0.263022	0.052714	0.167688	0.0259767	0.159146	0.156619	0.159146	0.159146	0	0.164662	0.167688
Ref_*K. pastoris*	0.263022	0.145727	0.0374354	0.182269	0.0751681	0.0770708	0.0025468	0.00274254	0.164662	0	0.0770708
Ref_*K. phaffii*	0.263022	0.151946	0.0786628	0.161822	0.0182524	0.00420918	0.0774628	0.0778587	0.167688	0.0770708	0

C. matrit. = Citeromyces matritensis, K. mondav. = *K. mondaviorum*, *K. pseudopast*. = *K. pseudopastoris*, *K. phaffii* (2) = *K. phaffii* UWOPS 03-328y3,
K. pastoris (2) = *K. pastoris* DSMZ 70877, *K. pastoris* (3) = *K. pastoris* CBS 9178, Ref_*K. pastoris* = *K. pastoris* CBS 704, Ref_*K. phaffii* = *K. phaffii* CBS 7435

Table S2: Orthologs of *Scheffersomyces stipitis* xylose reductase (XR) in different *Komagataella* species. Start and Stop refer to the nucleotide position of each gene in the genome assembly. Seq id gives the % sequence identity of the translated ORF to the *S. stipitis* protein.

**Strain**	**Contig**	**Start**	**Stop**	**DNA strand**	**Seq id [%]**	**Protein length**	**Annotation in *K. phaffii* CBS 7435**
K. mondaviorum CBS 15017	contig5	680262	679303	minus	68.15	319	Gre3
	contig2	289047	289977	plus	36.79	309	Ypr1
	contig1	2254062	2254907	plus	32.39	281	PP7435_Chr2-0714
	contig1	1034828	1033929	minus	30.65	299	PP7435_Chr4-0551
K. ulmi CBS 12361	contig3	1026994	1026035	minus	67.87	320	Gre3
	contig1	1253391	1254320	plus	36.16	309	Ypr1
	contig2	1355033	1355878	plus	33.33	281	PP7435_Chr2-0714
	contig4	640886	639987	minus	29.17	299	PP7435_Chr4-0551
K. populi CBS 12362	contig4	818460	819419	plus	67.62	319	Gre3
	contig5	474100	473171	minus	36.8	309	Ypr1
	contig2	845514	844669	minus	32.7	281	PP7435_Chr2-0714
	contig1	1800090	1800989	plus	29.97	299	PP7435_Chr4-0551
K. kurtzmanii CBS 12817	contig2	1412680	1411721	minus	67.54	319	Gre3
	contig3	25157	26086	plus	36.36	309	Ypr1
	contig1	1344402	1345247	plus	33.65	281	PP7435_Chr2-0714
	contig4	382089	382988	plus	29.49	299	PP7435_Chr4-0551
K. phaffii UWOPS 03-328y3	contig2	1446336	1445377	minus	67.54	319	Gre3
	contig1	1235528	1236457	plus	36.99	309	Ypr1
	contig3	1065727	1064882	minus	33.96	281	PP7435_Chr2-0714
	contig4	345894	344995	minus	29.11	299	PP7435_Chr4-0551
K. pastoris DSMZ 70877	contig3	825331	826290	plus	68.53	319	Gre3
	contig1	2099022	2098093	minus	36.79	309	Ypr1
	contig2	1369684	1370529	plus	33.33	281	PP7435_Chr2-0714
	contig4	939156	940055	plus	28.80	299	PP7435_Chr4-0551
K. pastoris CBS 9178	contig3	1027767	1026808	minus	68.2	319	Gre3
	contig1	2079669	2078740	minus	36.79	309	Ypr1
	contig2	1336610	1335765	minus	33.33	281	PP7435_Chr2-0714
	contig4	939592	940491	plus	28.80	299	PP7435_Chr4-0551
K. pseudopastoris CBS 9187	contig2	1279287	1278328	minus	67.83	319	Gre3
	contig2	293297	294226	plus	37.12	309	Ypr1
	contig4	836018	835173	minus	32.39	281	PP7435_Chr2-0714
	contig1	993828	992929	minus	29.97	299	PP7435_Chr4-0551

Table S3: Orthologs of *S. stipitis* xylitol dehydrogenase (XDH) in different *Komagataella* species. Start and Stop refer to the nucleotide position of each gene in the genome assembly. Seq id gives the % sequence identity of the translated ORF to the *S. stipitis* protein.

Strain	Contig	Start	Stop	DNA strand	Seq id [%]	Protein length	Annotation in *K. phaffii* CBS 7435
K. mondaviorum CBS 15017	contig2	169306	170352	plus	54.25	348	Sor1
K. ulmi CBS 12361	contig1	1115051	1116097	plus	54.8	348	Sor1
K. populi CBS 12362	contig1	2634432	2633386	minus	53.7	348	Sor1
K. kurtzmanii CBS 12817	contig5	1095113	1096159	plus	54.52	348	Sor1
K. phaffii UWOPS 03-328y3	contig1	1097155	1098201	plus	54.8	348	Sor1
K. pastoris DSMZ 70877	contig1	2237414	2236368	minus	54.52	409	Sor1
K. pastoris CBS 9178	contig1	2218021	2216975	minus	54.52	409	Sor1
K. pseudopastoris CBS 9187	contig1	158944	159990	plus	53.97	348	Sor1

Table S4: Orthologs of *S. stipitis* xylulokinase (XKS) in different *Komagataella* species. Start and Stop refer to the nucleotide position of each gene in the genome assembly. Seq id gives the % sequence identity of the translated ORF to the *S. stipitis* protein.

Strain	Contig	Start	Stop	DNA strand	Seq id [%]	Protein length	Annotation in *K. phaffii* CBS 7435
K. mondaviorum CBS 15017	contig2	170770	172593	plus	49.76	607	Xks1
K. ulmi CBS 12361	contig1	1116531	1118348	plus	49.44	605	Xks1
K. populi CBS 12362	contig1	2632967	2631144	minus	50.08	607	Xks1
K. kurtzmanii CBS 12817	contig5	1096552	1098405	plus	49.28	617	Xks1
K. phaffii UWOPS 03-328y3	contig1	1098596	1100449	plus	49.76	617	Xks1
K. pastoris DSMZ 70877	contig1	2235937	2234120	minus	50.08	605	Xks1
K. pastoris CBS 9178	contig1	2216544	2214727	minus	50.24	605	Xks1
K. pseudopastoris CBS 9187	contig1	160409	162232	plus	49.84	607	Xks1

A)

```
S.stipitis_CBS6054_XR          -MPSIKLNSGYDMPAVGFGCWKVDVDTCSEQIYRAIKTGYRLFDGAEDYANEKLVGAGVK      59
K.populi_CBS12362              MASVLKLNNGLKLPQVGLGVWKIPNELTAETVYNAIKQGYRLFDGAEDYGNEKEVGQGVR      60
K.pseudopastoris_CBS9187       MASVLKLNNGLKLPQVGLGVWKIPNELTAETVYNAIKQGYRLFDGAEDYGNEKEVGQGVR      60
K.mondaviorum_CBS15017         MASLLTLNNGLKLPQVGLGVWKIPNELTAETVYNAIKQGYRLFDGAEDYGNEKEVGQGVR      60
K.kurtzmanii_CBS12817          MATLLKLNNGLKLPQVGLGVWKIPNELTAETVYNAIKQGYRLFDGAEDYGNEKEVGQGVR      60
K.phaffii_UWOPS03-328y3        MATLLKLNNGLKLPQVGLGVWKIPNELTAETVYNAIKQGYRLFDGAEDYGNEKEVGQGVR      60
K.phaffii_CBS7435              MATLLKLNNGLKLPQVGLGVWKIPNELTAETVYNAIKQGYRLFDGAEDYGNEKEVGQGVR      60
K.ulmi_CBS12361                MASLLKLNNGLKLPQVGLGVWKIPNELTAETVYNAIKQGYRLFDGAEDYGNEKEVGQGVR      60
K.pastoris_DSMZ70877           MATLLKLNNGLKLPQVGLGVWKIPNELTAETVYNAIKQGYRLFDGAEDYGNEKEVGQGVR      60
K.pastoris_CBS9178             MATLLKLNNGLKLPQVGLGVWKIPNELTAETVYNAIKQGYRLFDGAEDYGNEKEVGQGVR      60
K.pastoris_CBS704              MATLLKLNNGLKLPQVGLGVWKIPNELTAETVYNAIKQGYRLFDGAEDYGNEKEVGQGVR      60
                                   :.**.* .:* **:* **:  :  :* :*.*** ***********.*** ** **:

S.stipitis_CBS6054_XR          KAIDEGIVKREDLFLTSKLWNNYHHPDNVEKALNRTLSDLQVDYVDLFLIHFPVTFKFVP      119
K.populi_CBS12362              KAIDEGIVKREDLFIVSKLWNNYHHPDNVEKGLDRTLSDLGLDYLDLFYIHFPIAFKFVP      120
K.pseudopastoris_CBS9187       KAIDEGIVKREDLFIVSKLWNNYHHPDNVEKALDRTLSDLGLDYLDLFYIHFPIAFKFVP      120
K.mondaviorum_CBS15017         KAIDEGIVKREDLFIVSKLWNNYHHPDNVGKALDRTLSDLGLDYLDLFYIHFPIAFKFVP      120
K.kurtzmanii_CBS12817          RAIDEGLVKREDLFIVSKLWNNYHHPDNVGKALDRTLSDLGLDYLDLFYIHFPIAFKFVP      120
K.phaffii_UWOPS03-328y3        RAIDEGLVKREDLFIVSKLWNNYHHPDNVGKALDRTLSDLGLDYLDLFYIHFPIAFKFVP      120
K.phaffii_CBS7435              RAIDEGLVKREDLFIVSKLWNNYHHPDNVGKALDRTLSDLGLDYLDLFYIHFPIAFKFVP      120
K.ulmi_CBS12361                KAIDEGLVKREDLFIVSKLWNNYHHPDNVAKALDRTLSDLGLDYLDLYYIHFPIAFKFVP      120
K.pastoris_DSMZ70877           KAIDEGLVKREDLFIVSKLWNNYHHPDNVAKALDRTLSDLGLDYLDLYYIHFPIAFKFVP      120
K.pastoris_CBS9178             KAIDEGLVKREDLFIVSKLWNNYHHPENVAKALDRTLSDLGLDYLDLYYIHFPIAFKFVP      120
K.pastoris_CBS704              KAIDEGLVKREDLFIVSKLWNNYHHPENVAKALDRTLSDLGLDYLDLYYIHFPIAFKFVP      120
                               :*****:*******:.**********:** *.*:****** :**:**: ****::*****

S.stipitis_CBS6054_XR          LEEKYPPGFYCGKGDNFDYEDVPILETWKALEKLVKAGKIRSIGVSNFPGALLLDLLRGA      179
K.populi_CBS12362              LEEKYPPGFYCGDGDNFHYEDVPLIDTYRALEKLVDAGKIKSLGVSNFTGALLQDLLRGA      180
K.pseudopastoris_CBS9187       LEEKYPPAFYCGDGDNFHYEDVPLIDTYRALEKLVDAGKIKSLGVSNFTGALLQDLLRGA      180
K.mondaviorum_CBS15017         LEEKYPPAFYCGDGNNFHYEDVPLSDTYRALEKLVDAGKIRSLGVSNFNGALLQDLLRGV      180
K.kurtzmanii_CBS12817          LEEKYPPAFYCGDGNNFHYEDVPLLDTYRALERLVDAGRIKSLGVSNFNGALLQDLLRGA      180
K.phaffii_UWOPS03-328y3        LEEKYPPAFYCGDGNNFHYEDVPLLDTYRALERLVDAGRIKSLGVSNFNGALLQDLLRGA      180
K.phaffii_CBS7435              LEEKYPPAFYCGDGNNFHYEDVPLLDTYRALERLVDAGRIKSLGVSNFNGALLQDLLRGA      180
K.ulmi_CBS12361                LEEKYPPGFYCGDGNKFHYEDVPLLDTYRALEKLVDAGRIKSLGVSNFNGALLQDLLRGA      180
K.pastoris_DSMZ70877           LEEKYPPGFYCGDGNNFHYEDVPLLDTYRALEKLVDAGRIKSLGVSNFNGALLQDLLRGA      180
K.pastoris_CBS9178             LEEKYPPGFYCGDGNNFHYEDVPLLDTYRALEKLVDAGRIKSLGVSNFNGALLQDLLRGA      180
K.pastoris_CBS704              LEEKYPPGFYCGDGNNFHYEDVPLLDTYRALEKLVDAGRIKSLGVSNFNGALLQDLLRGA      180
                               *******.****.*::*.*****: :*::***:**.**:*:*:***** **** *****.

S.stipitis_CBS6054_XR          TIKPSVLQVEHHPYLQQPRLIEFAQSRGIAVTAYSSFGPQSFVELNQGRALNTSPLFENE      239
K.populi_CBS12362              RIRPAALQIEHHPYLVQQNLIDYAQSEDIVVVAYSSFGPQSFLELKFTRALDAVSLFEHD      240
K.pseudopastoris_CBS9187       RIKPAALQIEHHPYLVQQNLIDYAQSEDIVVVAYSSFGPQSFLELKLTRALDAVSLFEHD      240
K.mondaviorum_CBS15017         RIKPVALQIEHHPYLVQQNLIEYAQSEDIVVVAYSSFGPQSFLELNVTKALNAVSLFEHD      240
K.kurtzmanii_CBS12817          RIKPVALQIEHHPYLVQQKLIEYAQSEDIVVVAYSSFGPQSFLELKVNKALTAVSLFEHD      240
K.phaffii_UWOPS03-328y3        RIKPVALQIEHHPYLVQQKLIEYAQSEDIVVVAYSSFGPQSFLELKVNKALTAVSLFEHD      240
K.phaffii_CBS7435              RIKPVALQIEHHPYLVQQKLIEYAQSEDIVVVAYSSFGPQSFLELKVNKALTAVSLFEHD      240
K.ulmi_CBS12361                RIKPVALQIEHHPYLVQQKLIEYAQSEDIVVVAYSSFGPQSFLELKVNKALTAVSLFEHD      240
K.pastoris_DSMZ70877           RIKPVALQIEHHPYLVQQKLIEYAQSEDIVVVAYSSFGPQSFLELKVNKALTAVSLFEHD      240
K.pastoris_CBS9178             RIKPVALQIEHHPYLVQQKLIEYAQSEDIVVVAYSSFGPQSFLELKVNKALTAVSLFEHD      240
K.pastoris_CBS704              RIKPVALQIEHHPYLVQQKLIEYAQSEDIVVVAYSSFGPQSFLELKVNKALTAVSLFEHD      240
                                *:* .**:****** * .**::***..*.*.**********:**:  :** :  ***::

S.stipitis_CBS6054_XR          TIKAIAAKHGKSPAQVLLRWSSQRGIAIIPKSNTVPRLLENKDVNSFDLDEQDFADIAKL      299
K.populi_CBS12362              TIKRIAQSHNRSTGEVLLRWATQRGLAVIPKSSKPDRLLSNLHVDSFDLTKEDLEAISAL      300
K.pseudopastoris_CBS9187       TIKRIAQSHNRSTGEVLLRWATQRGLAVIPKSSKPDRLTSNLHIDSFDLTKEDLEAISAL      300
K.mondaviorum_CBS15017         TIKQIAQAHNRSAGEVLLRWATQRGLAIIPKSSKPDRLTSNLHINSFDLTKEDLEAISSL      300
K.kurtzmanii_CBS12817          VIKKIAQAHNRSAGEVLLRWATQRGLAIIPKSSKPERLSSNLHINSFDLTKEDLETISSL      300
K.phaffii_UWOPS03-328y3        VIKKIAQAHNRSAGEVLLRWATQRGLAIIPKSSKPERLSSNLHINSFDLTKEDLETISSL      300
K.phaffii_CBS7435              VIKKIAQAHNRSAGEVLLRWATQRGLAIIPKSSKPERLSSNLHINSFDLTKEDLETISSL      300
K.ulmi_CBS12361                VIKKIAQAHNRSAGEVLLRWATQRGLAIIPKSSKPERLTSNLHINSFDLTQEDLETISSL      300
K.pastoris_DSMZ70877           VIKKIAQAHNKSAGEVLLRWATQRGLAIIPKSSKPERLTSNLHINSFDLTQEDLETISSL      300
K.pastoris_CBS9178             VIKKIAQAHNKSAGEVLLRWATQRGLAIIPKSSKPERLTSNLHINSFDLTQEDLETISSL      300
K.pastoris_CBS704              VIKKIAQAHNKSAGEVLLRWATQRGLAIIPKSSKPERLTSNLHINSFDLTQEDLETISSL      300
                               .** **  *.:* .:*****::***:*:****..  ** .* .::**** ::*:  *: *

S.stipitis_CBS6054_XR          DINLRFNDPWDWDKIPIFV*       318
K.populi_CBS12362              DLGLRFNDPWDWNKIPTFA*       319
K.pseudopastoris_CBS9187       DLGLRFNDPWDWNKIPIFA*       319
K.mondaviorum_CBS15017         NLGLRFNDPWDWDKIPIFA*       319
K.kurtzmanii_CBS12817          DLGLRFNDPWDWDKIPIFA*       319
K.phaffii_UWOPS03-328y3        DLGLRFNDPWDWDKIPIFA*       319
K.phaffii_CBS7435              DLGLRFNDPWDWDKIPIFA*       319
K.ulmi_CBS12361                DLGLRFNDPWDWDKIPIFA*       319
K.pastoris_DSMZ70877           DLGLRFNDPWDWDKIPIFA*       319
K.pastoris_CBS9178             DLGLRFNDPWDWDKIPIFA*       319
K.pastoris_CBS704              DLGLRFNDPWDWDKIPIFA*       319
                               ::.*********:*** *.*
```

B)

```
S.stipitis_CBS6054_XDH          ------------------------------------------------------------    0
K.pseudopastoris_CBS9187        ------------------------------------------------------------    0
K.mondaviorum_CBS15017          ------------------------------------------------------------    0
K.populi_CBS12362               ------------------------------------------------------------    0
K.ulmi_CBS12361                 ------------------------------------------------------------    0
K.pastoris_DSMZ70877            MLLVFFTSDDCAGLKNSCIPEKLSQTYVPHVIPSSSTKEFKTYSLPNNLSLFPLYFIHYR    60
K.pastoris_CBS9178              MLLVFFTSDDCAGLKNSCIPEKLSQTYVPHVIPSSSTKEFKTYSLPNNLSLFPLYFIHYR    60
K.pastoris_CBS704               MLLVFFTSDDCAGLKNSCIPEKLSQTYVPHVIPSSSTKEFKTYSLPNNLSLFPLYFIHYR    60
K.kurtzmanii_CBS12817           ------------------------------------------------------------    0
K.phaffii_UWOPS03-328y3         ------------------------------------------------------------    0
K.phaffii_CBS7435               ------------------------------------------------------------    0

S.stipitis_CBS6054_XDH          -MTANPSLVLNKIDDISFETYDAPEISEPTDVLVQVKKTGICGSDIHFYAHGRIGNFVLT    59
K.pseudopastoris_CBS9187        -MSGNPSVVLKKVNEIVIEDRPVPDIADPHYVKIAIKKTGICGSDVHFYTDGCCGSFKLE    59
K.mondaviorum_CBS15017          -MSDNPSVILKKVNEIVIEDRPVPDIADPHYVKIAIKKTGICGSDVHFYTDGCCGSFKLE    59
K.populi_CBS12362               -MSDNPSVVLKKVNEIVIEDRPVPDIADPHYVKIAIKKTGICGSDVHFYTDGCCGSFKLE    59
K.ulmi_CBS12361                 -MSDNPSVVLKKINEIVIEDRPIPAVEDPHYVKIAIKKTGICGSDVHFYTDGCCGSFKLE    59
K.pastoris_DSMZ70877            NMSDNPSVVLKKINEIVIEDRPIPAIEDPHYVKIAIKKTGICGSDVHFYTDGCCGSFKLE    120
K.pastoris_CBS9178              NMSDNPSVVLKKINEIVIEDRPIPAIEDPHYVKIAIKKTGICGSDVHFYTDGCCGSFKLE    120
K.pastoris_CBS704               NMSDNPSVVLKKINEIVIEDRPIPAIEDPHYVKVAIKKTGICGSDVHFYTDGCCGSFKLE    120
K.kurtzmanii_CBS12817           -MSDNPSVILKKISEIVIEDRPIPAIEDPHYVKIAIKKTGICGSDVHFYTDGCCGSFKLE    59
K.phaffii_UWOPS03-328y3         -MSDNPSVILKRINEIVIEDRPIPAIEDPHYVKIAIKKTGICGSDVHFYTDGCCGSFKLE    59
K.phaffii_CBS7435               -MSDNPSVILKRINEIVIEDRPIPAIEDPHYVKIAIKKTGICGSDVHFYTDGCCGSFKLE    59
                                 *: ***::*:::.:* :*    * : :*  * : :*********:***:.*  *.* *

S.stipitis_CBS6054_XDH          KPMVLGHESAGTVVQVGKGVTSLKVGDNVAIEPGIPSRFSDEYKSGHYNLCPHMAFAATP    119
K.pseudopastoris_CBS9187        SPMVLGHESSGIVVEVGSEVKSLKVGDKVACEPGIPSRYSDAYKSGHYNLCPEMAFAATP    119
K.mondaviorum_CBS15017          SPMVLGHESSGIVVEVGSEVKSLKVGDKVACEPGIPSRYSDAYKSGHYNLCPEMAFAATP    119
K.populi_CBS12362               SPMVLGHESSGIVVEVGSEVKSLKVGDKVACEPGIPSRYSGAYKSGHYNLCPEMAFAATP    119
K.ulmi_CBS12361                 SPMVLGHESAGIVVEVGSDVKSLKVGDKVACEPGIPSRYSNAYKSGHYNLCPEMAFAATP    119
K.pastoris_DSMZ70877            SPMVLGHESAGIVVEVGSDVKSLKVGDKVACEPGIPSRYSNAYKSGHYNLCPEMAFAATP    180
K.pastoris_CBS9178              SPMVLGHESAGIVVEVGSDVKSLKVGDKVACEPGIPSRYSNAYKSGHYNLCPEMAFAATP    180
K.pastoris_CBS704               SPMVLGHESAGIVVEVGSDVKSLKVGDKVACEPGIPSRYSNAYKSGHYNLCPEMAFAATP    180
K.kurtzmanii_CBS12817           SPMVLGHESAGIVVEVGSEVKSLKIGEKVACEPGIPSRYSNAYKSGHYNLCPEMAFAATP    119
K.phaffii_UWOPS03-328y3         SPMVLGHESAGIVVEVGSEVKSLRVGDKVACEPGIPSRYSNAYKSGHYNLCPEMAFAATP    119
K.phaffii_CBS7435               SPMVLGHESAGIVVEVGSEVKSLRVGDKVACEPGIPSRYSNAYKSGHYNLCPEMAFAATP    119
                                .********:* **:**. *.**::*::** *******:*. **********.*******

S.stipitis_CBS6054_XDH          NSKEGEPNPPGTLCKYFKSPEDFLVKLPDHVSLELGALVEPLSVGVHASKLGSVAFGDYV    179
K.pseudopastoris_CBS9187        P-------IDGTLCRYYLLPEDFCVKLPEHVSLEEGALVEPLSVAVHAARLAKITFGDSV    172
K.mondaviorum_CBS15017          P-------IDGTLCRYFLLPEDFCVKLPEHVSLEEGALVEPLSVAVHAARLAKITFGDSV    172
K.populi_CBS12362               P-------IDGTLCRYYLLPEDFCVKLPEHVSLEEGALAEPLSVAVHAARLAKITFGDSV    172
K.ulmi_CBS12361                 P-------IDGTLCRYYLLPEDFCVKLPDHVSLEEGALVEPLSVAVHAARLAKITFGDSV    172
K.pastoris_DSMZ70877            P-------IDGTLCRYYLLPEDFCVKLPGHVSLEEGALVEPLSVAVHAARLAKITFGDSV    233
K.pastoris_CBS9178              P-------IDGTLCRYYLLPEDFCVKLPGHVSLEEGALVEPLSVAVHAARLAKITFGDSV    233
K.pastoris_CBS704               P-------IDGTLCRYYLLPEDFCVKLPGHVSLEEGALVEPLSVAVHAARLAKITFGDSV    233
K.kurtzmanii_CBS12817           P-------IDGTLCRYFLLPEDFCVKLPEHVSLEEGALVEPLSVAVHAARLAKITFGDSV    172
K.phaffii_UWOPS03-328y3         P-------IDGTLCRYFLLPEDFCVKLPEHVSLEEGALVEPLSVAVHAARLAKITFGDSV    172
K.phaffii_CBS7435               P-------IDGTLCRYFLLPEDFCVKLPEHVSLEEGALVEPLSVAVHAARLAKITFGDSV    172
                                          ****:*:  **** **** ***** ***.*****.***::*..::*** *

S.stipitis_CBS6054_XDH          AVFGAGPVGLLAAAVAKTFGAKGVIVVDIFDNKLKMAKDIGA-ATHTFNSKTGGSEELIK    238
K.pseudopastoris_CBS9187        VVFGAGPVGLLVAATARAYGATSVLIVDISDDKLTLAKDTLKVATQSFNS-KNGMDNLAD    231
K.mondaviorum_CBS15017          VVFGAGPVGLLVAATARAYGATSVLIVDISDDKLTLAKDTLKVATQSFNS-KNGMDNLSD    231
K.populi_CBS12362               VVFGAGPVGLLVAATARAYGATSVLIVDISDDKLTLAKDTLKVATQSFNS-KNGMDNLAD    231
K.ulmi_CBS12361                 VVFGAGPVGLLVAATARAYGATKVLIVDIFDDKLTLATDTLHVATHSYNS-KNGMGNLLE    231
K.pastoris_DSMZ70877            VVFGAGPVGLLVAATARAYGATKVLIVDIFDDKLTLATDTLHVATNSYNS-KNGMDNLLE    292
K.pastoris_CBS9178              VVFGAGPVGLLVAATARAYGATKVLIVDIFDDKLTLATDTLHVATNSYNS-KNGMDNLLE    292
K.pastoris_CBS704               VVFGAGPVGLLVAATARAYGATKVLIVDIFDDKLTLATDTLHVATNSYNS-KNGMDNLLE    292
K.kurtzmanii_CBS12817           VVFGAGPVGLLVAATARAYGATNVLIVDIFDDKLTLAKDTLQVATHSFNS-KNGMDNLLE    231
K.phaffii_UWOPS03-328y3         VVFGAGPVGLLVAATAKAYGATNVLIVDIFDDKLTLAKDTLQVATHSFNS-KNGMDNLLE    231
K.phaffii_CBS7435               VVFGAGPVGLLVAATARAYGATNVLIVDIFDDKLTLAKDTLQVATHSFNS-KNGMDNLLE    231
                                .**********.**.*:::**. *::*** *:**.:*.*    **:::** ..*  :* .

S.stipitis_CBS6054_XDH          AFGGNVPNVVLECTGAEPCIKLGVDAIAPGGRFVQVGNAAG-PVSFPITVFAMKELTLFG    297
K.pseudopastoris_CBS9187        ALEGKRPNISIDCTGVESCIAAGIKALAPRGIHVQVGMGKAEYNKFPLGLVCEKECTVEG    291
K.mondaviorum_CBS15017          ALEGKHPNVSIDCTGVESCIAAGIKALAPRGIHVQVGMGKAEYNNFPLGLICEKECTVKG    291
K.populi_CBS12362               ALEGKHPNISIDCTGVESCIAAGIKALAPRGIHVQVGMGKAEYNNFPLGLICEKECTVKG    291
K.ulmi_CBS12361                 SFEGKRPNVSIDCTGVESCIAAGINALAPRGVHVQVGMGKSEYNNFPLGVICEKECIVKG    291
K.pastoris_DSMZ70877            SFEGKHPNVSIDCTGVESCIAAGINALAPRGVHVQVGMGKSEYNNFPLGVICEKECIVKG    352
K.pastoris_CBS9178              SFEGKHPNVSIDCTGVESCIAAGINALAPRGVHVQVGMGKSEYNNFPLGVICEKECIVKG    352
K.pastoris_CBS704               SFEGKHPNVSIDCTGVESCIAAGINALAPRGVHVQVGMGKSEYNNFPLGVICEKECIVKG    352
K.kurtzmanii_CBS12817           SFEGKHPNVSIDCTGVESCIAAGINALAPRGVHVQVGMGKSEYNNFPLGLICEKECIVKG    291
K.phaffii_UWOPS03-328y3         SFEGKHPNVSIDCTGVESCIAAGINALAPRGVHVQVGMGKSEYNNFPLGLICEKECIVKG    291
K.phaffii_CBS7435               SFEGKHPNVSIDCTGVESCIAAGINALAPRGVHVQVGMGKSEYNNFPLGLICEKECIVKG    291
                                :: *: **: ::***.* **  *:.*:** * .**** . .   .**: :.. **  : *
```

```
S.stipitis_CBS6054_XDH         SFRYGFNDYKTAVGIFDTNYQNGRENAPIDFEQLITHRYKFKDAIEAYDLVRAGKGAVKC        357
K.pseudopastoris_CBS9187       VFRYCYNDYNLAVELIAS--------GKVDVKGVVTHRFKFTDSVDAYETVKQGK-AIKA        342
K.mondaviorum_CBS15017         VFRYCYNDYNLAVELIAS--------GKVDVKGLVTHRFKFTESVDAYETVKQGK-AIKA        342
K.populi_CBS12362              VFRYCWNDYNLAVELIAS--------GKVDVKGLVTHRFKFTDSVDAYETVKQGK-AIKA        342
K.ulmi_CBS12361                VFRYCYNDYNLAVELIAS--------GKVEVKGLVTHRFKFTEAVDAYETVRQGK-AIKA        342
K.pastoris_DSMZ70877           VFRYCYNDYNLAVELIAS--------GKVEVKGLVTHRFKFTEAVDAYETVRQGK-AIKA        403
K.pastoris_CBS9178             VFRYCYNDYNLAVELIAS--------GKVEVKGLVTHRFKFTEAVDAYETVRQGK-AIKA        403
K.pastoris_CBS704              VFRYCYNDYNLAVELIAS--------GKVEVKGLVTHRFKFTEAVDAYETVRQGK-AIKA        403
K.kurtzmanii_CBS12817          VFRYCYNDYNLAVELIAS--------GKVEVKGLVTHRFKFTEAVDAYETVRQGK-AIKA        342
K.phaffii_UWOPS03-328y3        VFRYCYNDYNLAVELIAS--------GKVEVKGLVTHRFKFTEAVDAYETVRQGK-AIKA        342
K.phaffii_CBS7435              VFRYCYNDYNLAVELIAS--------GKVEVKGLVTHRFKFTEAVDAYDTVRQGK-AIKA        342
                                *** :***: ** :: :        . ::.: ::***:**.::::**: *: ** *:*.

S.stipitis_CBS6054_XDH         LIDGPE* 363
K.pseudopastoris_CBS9187       IIDGPE* 348
K.mondaviorum_CBS15017         IIDGPE* 348
K.populi_CBS12362              MIDGPE* 348
K.ulmi_CBS12361                IIDGPE* 348
K.pastoris_DSMZ70877           IIDGPE* 409
K.pastoris_CBS9178             IIDGPE* 409
K.pastoris_CBS704              IIDGPE* 409
K.kurtzmanii_CBS12817          IIDGPE* 348
K.phaffii_UWOPS03-328y3        IIDGPE* 348
K.phaffii_CBS7435              IIDGPE* 348
                               :******
```

C)

```
S.stipitis_CBS6054_XKS         ---------MTTTPFDAPDKLFLGFDLSTQQLKIIVT-DENLAALKTYNVEFDSINSSVQ        50
K.mondaviorum_CBS15017         ------------MTESVPNALYLGFDLSTQQLKITSFEGCSLTHFRTYRVDFDKELSSYG        48
K.populi_CBS12362              ------------MSESVPNALYLGFDLSTQQLKITSFEGCSLTHFRTYRVDFDKEMSSYG        48
K.pseudopastoris_CBS9187       ------------MSESVPDALYLGFDLSTQQLKITSFEGCSLTHFRTYRVDFDEELSSYG        48
K.kurtzmanii_CBS12817          MVTKEIQNRDSALTESVPNDLYLGFDLSTQQLKITSFEGRSLTHFKTYRVDFDEELSVYG        60
K.phaffii_UWOPS03-328y3        MVTKEIQNRDSALTESVPNDLYLGFDLSTQQLKITSFEGRSLTHFKTYRVDFDEELSVYG        60
K.phaffii_CBS7435              MVTKEIQNRDSALTESVPNDLYLGFDLSTQQLKITSFEGRSLTHFKTYRVDFDEELSVYG        60
K.ulmi_CBS12361                ------------MSDSVPSDLYLGFDLSTQQLKITSFEGRSLTHFKTYRVDFDEELSVYG        48
K.pastoris_DSMZ70877           ------------MSALVPSDLYLGFDLSTQQLKITSFEGRSLTHFKTYRVDFDEELSAYG        48
K.pastoris_CBS9178             ------------MSALVPSDLYLGFDLSTQQLKITSFEGRSLTHFKTYRVDFDEELSAYG        48
K.pastoris_CBS704              ------------MSALVPSDLYLGFDLSTQQLKITSFEGRSLTHFKTYRVDFDEELSAYG        48
                                               .*. *:************    . .*: ::**.*:**.  *

S.stipitis_CBS6054_XKS         --KGVIAINDEISKGAIISPVYMWLDALDHVFEDMKKDGFPFNKVVGISGSCQQHGSVYW        108
K.mondaviorum_CBS15017         INNGVY---VTEESGEINAPVAMWIEALDLIFSKMQEDNFPFRNVKGMSGSCQQHGSVYW        105
K.populi_CBS12362              INNGVH---VTEETGEINAPVAMWIEALDLIFSKMQKDNFPFRNVKGMSGSCQQHGSVYW        105
K.pseudopastoris_CBS9187       INNGVY---VTEETGEINAPVAMWIEALDLIFSKMQKDNFPFRNVKGMSGSCQQHGSVYW        105
K.kurtzmanii_CBS12817          INNGVY---VNEETGEINAPVAMWVEALDLIFSKMQKDKFPFGNVKGMSGSCQQHGSVYW        117
K.phaffii_UWOPS03-328y3        INNGVY---VNEETGEINAPVAMWVEALDLIFSKMQKDKFPFGNVKGMSGSCQQHGSVYW        117
K.phaffii_CBS7435              INNGVY---VNEETGEINAPVAMWVEALDLIFSKMQKDKFPFGIVKGMSGSCQQHGSVYW        117
K.ulmi_CBS12361                IKNGVY---VNEETGEINAPVAMWIEALDLIFSKMQMDKFPFGNVKGMSGSCQQHGSVYW        105
K.pastoris_DSMZ70877           IKNGVY---VNEEIGEINAPVAMWIEALDLIFSKMQKDKFPFGNVKGMSGSCQQHGSVYW        105
K.pastoris_CBS9178             IKNGVY---VNEEIGEINAPVAMWIEALDLIFSKMQKDKFPFGNVKGMSGSCQQHGSVYW        105
K.pastoris_CBS704              IKNGVY---VNEEIGEINAPVAMWIEALDLIFSKMQKDKFPFGNVKGMSGSCQQHGSVYW        105
                                 :**       . * * :** **::*** :*..*: * ***  * *:************

S.stipitis_CBS6054_XKS         SRTAEKVLSELDAESSLSSQM-RSAFTFKHAPNWQDHSTGKELEEFERVIGA-DALADIS        166
K.mondaviorum_CBS15017         SNDAPDLLSSLSPSKDLKSQLCPEAFTFEMSPNWQDHSTGEELEIFERKAGSPDKLSKIT        165
K.populi_CBS12362              SKDAPDLLSSLSSSKDLKSQLCPEAFTFEMSPNWQDHSTVEELEIFERKAGSPDKLSKIT        165
K.pseudopastoris_CBS9187       SKDAPDFLSSLSSSKDLKSQLCPEAFTFGMSPNWQDHSTGEELEIFERKAGSPDKLSKIT        165
K.kurtzmanii_CBS12817          SKDAPDLLSSLSPSKDLKSQLCPKAFTFEKSPNWQDHSTGEELEIFERKAGSPENLSKIT        177
K.phaffii_UWOPS03-328y3        SKDAPDLLSSLSPSKDLKSQLCPKAFTFEKSPNWQDHSTGEELEIFERKAGSPENLSKIT        177
K.phaffii_CBS7435              SKDAPDLLSSLSPSKDLKSQLCPKAFTFEKSPNWQDHSTGEELEIFERKAGSPENLSKIT        177
K.ulmi_CBS12361                SKYAPDLLSSLSPLKDLKSQLCPKAFTFEKSPNWQDHSTGEELEIFERKAGSPENLSKIT        165
K.pastoris_DSMZ70877           SKDAPDLLSSLSPLKDLKSQLCPKAFTFEKSPNWQDHSTGEELEIFERKAGSPENLSKIT        165
K.pastoris_CBS9178             SKDAPDLLSSLSPLKDLKSQLCPKAFTFEKSPNWQDHSTGEELEIFERKAGSPENLSKIT        165
K.pastoris_CBS704              SKDAPDLLSSLSPLKDLKSQLCPKAFTFEKSPNWQDHSTGEELEIFERKAGSPENLSKIT        165
                               *. * ..**.*.  ..*.**:  .****  :******** :*** ***  *: : *:.*:

S.stipitis_CBS6054_XKS         GSRAHYRFTGLQIRKLSTRFKPEKYNRTARISLVSSFVASVLLGRITSIEEADACGMNLY        226
K.mondaviorum_CBS15017         GSRAHYRFTGPQIRKLAKRVNPELYKKTYRISLISSFLSSLLCGKITKIEESDGCGMNIY        225
K.populi_CBS12362              GSRAHYRFTGPQIRKLAKRVNPEQYKKTYRISLISSFLSSLLCGRITKIEESDGCGMNIY        225
K.pseudopastoris_CBS9187       GSRAHYRFTGPQIRKLAKRVNPEQYKKTYRISLISSFLSSLLCGKITKIEESDGCGMNIY        225
K.kurtzmanii_CBS12817          GSRAHYRFTGSQIRKLAKRVNPELYKETYRISLISSFLSSLLCGKITKIEESDGCGMNIY        237
K.phaffii_UWOPS03-328y3        GSRAHYRFTGSQIRKLAKRVNPELYKETYRISLISSFLSSLLCGKITKIEESDGCGMNIY        237
K.phaffii_CBS7435              GSRAHYRFTGSQIRKLAKRVNPELYKETYRISLISSFLSSLLCGRITKIEESDGCGMNIY        237
K.ulmi_CBS12361                GSRAHYRFTGSQIRKLAKRVNPELYKETYRISLISSFLSSLLCGKITKIEESDGCGMNIY        225
K.pastoris_DSMZ70877           GSRAHYRFTGSQIRKLAKRVNPELYKETYRISLISSFLSSLLCGKITKIEESDGCGMNIY        225
K.pastoris_CBS9178             GSRAHYRFTGSQIRKLAKRVNPELYKETYRISLISSFLSSLLCGKITKIEESDGCGMNIY        225
K.pastoris_CBS704              GSRAHYRFTGSQIRKLAKRVNPELYKETYRISLISSFLSSLLCGKITKIEESDGCGMNIY        225
                               ********** *****:.*.:** *:.* ****:***::*:* *:**.***:*.****:*
```

```
S.stipitis_CBS6054_XKS          DIEKREFNEELLAIAAGVHPELDGVEQDGEIYRAGINELKRKLGPVKPITYESEGDIASY    286
K.mondaviorum_CBS15017          DIQNSKYDENLLAISAAVDPEIDGATEDE--RQKGVTELKNKLQDLEPVGYHSIGSISAY    283
K.populi_CBS12362               DIQNSKYDEDLLAISAAVDPEIDGATEVE--REQGVTELKNKLQDLEPVGYHSIGSISAY    283
K.pseudopastoris_CBS9187        DIQNSKYDEDLLAISAAVDPEIDGATEDE--RQQGVTELKNKLQDLEPVGYHSIGSISAY    283
K.kurtzmanii_CBS12817           DIQNSRYDEDLLAVTAAVDPEIDGATEHE--RQEGVARLKDKLQDLEPVGFRSIGTISAY    295
K.phaffii_UWOPS03-328y3         DIQNSRYDEDLLAVTAAVDPEIDGATEHE--RQEGVARLKDKLQDLEPVGYRSIGTIAAY    295
K.phaffii_CBS7435               DIQNSRYDEDLLAVTAAVDPEIDGATEHE--RQEGVARLKDKLQDLEPVGYRSIGTIAAY    295
K.ulmi_CBS12361                 DIQNSRYSEELLAVTAAVDPEIDGATEDE--RQEGVARLKDKLQDLEPVGYRSIGSISAY    283
K.pastoris_DSMZ70877            DIQNSRYSEDLLAVTAAVDPKIDGATEDE--RREGVARLKDKLQDLEPVGYRSIGSISAY    283
K.pastoris_CBS9178              DIQNSRYSEDLLAVTAAVDPEIDGATEDE--RREGVARLKDKLQDLEPVGYRSIGSISAY    283
K.pastoris_CBS704               DIQNSRYSEDLLAVTAAVDPEIDGATEDE--RREGVARLKDKLQDLEPVGYRSIGSISAY    283
                                **:: .:.*:***::*.*.*::**. :     . *: .** **  ::*: :.* * *::*

S.stipitis_CBS6054_XKS          FVTRYGFNPDCKIYSFTGDNLATIISLPLAPNDALISLGTSTTVLIITKNYAPSSQYHLF    346
K.mondaviorum_CBS15017          FVEKYGFSSDTKVFSFTGDNLATILSLPLHNDDILVSLGTSTTVLLVTETYWPNPNYHVF    343
K.populi_CBS12362               FVEKYGFSSDAKVFSFTGDNLATILSLPLHNDDVLVSLGTSTTVLLVTETYWPNPNYHVF    343
K.pseudopastoris_CBS9187        FVEKYGFRSDAKVFSFTGDNLATILSLPLHNDDILVSLGTSTTLLLVTETYWPNPNYHVF    343
K.kurtzmanii_CBS12817           FVEKYGFSEDGKVFSFTGDNLATILSLPLHNDDILVSLGTSTTVLLVTETYWPNSNYHVF    355
K.phaffii_UWOPS03-328y3         FVEKYGFSEDSKVFSFTGDNLATILSLPLHNDDILVSLGTSTTVLLVTETYWPNSNYHVF    355
K.phaffii_CBS7435               FVEKYGFSEDSKVFSFTGDNLATILSLPLHNDDILVSLGTSTTVLLVTETYWPNSNYHVF    355
K.ulmi_CBS12361                 FVEKYGFSEDSKIFSFTGDNLATILSLPLHNDDILVSLGTSTTVLLVTETYWPNSNYHVF    343
K.pastoris_DSMZ70877            FVEKYGFSKDSKIFSFTGDNLATILSLPLHNDDILVSLGTSTTVLLVTETYWPNSNYHVF    343
K.pastoris_CBS9178              FVEKYGFSKDSKIFSFTGDNLATILSLPLHNDDILVSLGTSTTVLLVTETYWPNSNYHVF    343
K.pastoris_CBS704               FVEKYGFSKDSKIFSFTGDNLATILSLPLHNDDILVSLGTSTTVLLVTETYWPNSNYHVF    343
                                ** :***  * *::**********:****  :* *:*******:*::*:.* *. :**:*

S.stipitis_CBS6054_XKS          KHPTMPDHYMGMICYCNGSLAREKVRDEVNEKFNVEDKKSWDKFNEILDKSTDFN--NKL    404
K.mondaviorum_CBS15017          KHPTVPGSYMVMLCYVNGALARNQIKAELDEKYNVSDPNDWTKFNEILDNSKPLHGNREL    403
K.populi_CBS12362               KHPTVPGSYMVMLCYVNGALARNQIKAKLDEKYNVSDSNDWTKFNEILDNSKPLHGNKEL    403
K.pseudopastoris_CBS9187        KHPTVPGSYMVMLCYVNGALARNQIKAKLDAKYNVSDSNDWTKFNEILDNSKPLHGNKEL    403
K.kurtzmanii_CBS12817           KHPTVPGSYMVMLCYVNGALARNQIKASLDKKYNLSDPNDWTKFNEILDKSKPLHGKEEL    415
K.phaffii_UWOPS03-328y3         KHPTVPGSYMVMLCYVNGALARNQIKTSLDKKYNVSDPNDWTKFNEILDKSKPLHGKEEL    415
K.phaffii_CBS7435               KHPTVPGSYMVMLCYVNGALARNQIKTSLDKKYNVSDPNDWTKFNEILDKSKPLHGKEEL    415
K.ulmi_CBS12361                 KHPTVPGSYMVMLCYVNGALARNQIKSSLDKKYNVSDSNDWTKFNEILDQSKPLHGKKEL    403
K.pastoris_DSMZ70877            KHPTVPGSYMVMLCYVNGALARNQIKASLDEKYNVSDSNDWTKFNEILDKSKPLHGKKEL    403
K.pastoris_CBS9178              KHPTVPGSYMVMLCYVNGALARNQIKASLDEKYNVSDSNDWTKFNEILDKSKPLHGKKEL    403
K.pastoris_CBS704               KHPTVPGSYMVMLCYVNGALARNQIKASLDEKYNVSDSNDWTKFNEILDKSKPLHGKKEL    403
                                ****:*. ** *:** **:***:::: .:: *:*:.* :.* *******:*. ::  .:*

S.stipitis_CBS6054_XKS          GIYFPLGEIVPNAAAQIKRSVLNSKNEIVDVELGDKNWQPEDDVSSIVESQTLSCRLRTG    464
K.mondaviorum_CBS15017          GVYFPKGEIIPNCIAQTKRYLYDDTK----RELVAREWNIEDDVVSIVESQALSCRLRSG    459
K.populi_CBS12362               GVYFPKGEIIPNCIAQTKRHSYDDKK----KELVAREWKIEDDVVSIVESQALSCRLRSG    459
K.pseudopastoris_CBS9187        GVYFPKGEIIPNCIAQTKRYSYDDKK----KELVAREWKIEDDVVSIVESQALSCRLRSG    459
K.kurtzmanii_CBS12817           GVYFPKGEIIPNCVAQTKRFSYDAKS----KKLVTANWDIEDDVVSIVESQALSCRLRSG    471
K.phaffii_UWOPS03-328y3         GVYFPKGEIIPNCVAQTKRFSYDAKS----KKLVTANWDIEDDVVSIVESQALSCRLRSG    471
K.phaffii_CBS7435               GVYFPKGEIIPNCVAQTKRFSYDAKS----KKLVTANWDIEDDVVSIVESQALSCRLRSG    471
K.ulmi_CBS12361                 GVYFPKGEIIPNCIAQTKRYSYDVKS----KKLIAADWDIEDDVVSIVESQALSCRLRSG    459
K.pastoris_DSMZ70877            GVYFPKGEIIPNCIAQTKRYSYDAKS----RKLVAADWDIEDDAVSIVESQALSCRLRSG    459
K.pastoris_CBS9178              GVYFPKGEIIPNCIAQTKRYSYDAKS----RKLVAADWDIEDDAVSIVESQALSCRLRSG    459
K.pastoris_CBS704               GVYFPKGEIIPNCIAQTKRYSYDAKS----RKLVAADWDIEDDAVSIVESQALSCRLRSG    459
                                *:*** ***:**. ** **   : ..     :*   :*. ***. ******:******:*

S.stipitis_CBS6054_XKS          PMLSKSGDSSASSSASPQPEGDGTDLHKVYQDLVKKFGDLYTDGKKQTFESLTARPNRCY    524
K.mondaviorum_CBS15017          PLYHGSEEIDEVEK------------NE-VTKKLSVFSKISADGKDQRISDLISHPKKAF    506
K.populi_CBS12362               PLYHGSEEIDEVEK------------SE-VAKKLSTFSKISADGKDQRISDLISHPKKAF    506
K.pseudopastoris_CBS9187        PLYHGSEEIDEVEK------------SE-VTKKLSTFSKISADGKDQCIFDLISHPKKAF    506
K.kurtzmanii_CBS12817           PLYHGSDETDQEEE------------SE-VIQRLSNFPKISADGKDQRLPDLISHPKKAF    518
K.phaffii_UWOPS03-328y3         PLYHGSDETDQEEE------------SE-VIQRLSNFPKISADGKDQRLPDLISHPKKAF    518
K.phaffii_CBS7435               PLYHGSDETDQEEE------------SE-VIQRLSNFPKISADGKDQRLPDLISHPKKAF    518
K.ulmi_CBS12361                 PLYHGSDEIDEKEE------------NE-VTQRLSIFPKISADGKDQRLANLISHPKKAF    506
K.pastoris_DSMZ70877            PLYHGSDETDEKEE------------IE-VTRRLSIFPKISADGKDQRLADLISHPKKAF    506
K.pastoris_CBS9178              PLYHGSDETDEKEE------------IE-VTRRLSIFPKISADGKDQRLADLISHPKKAF    506
K.pastoris_CBS704               PLYHGSDETDEKEE------------IE-VTRRLSIFPKISADGKDQRLADLISHPKKAF    506
                                *:   * : .  ..             :     :. * .: :***.* : .* ::*::.:

S.stipitis_CBS6054_XKS          YVGGASNNGSIIRKMGSILAPVNGNYKVDIPNACALGGAYKASWSYECEAKKEWIGYDQY    584
K.mondaviorum_CBS15017          YVGGASQNVSIVRKFSEILGAKEGNYQINLGDACAIGGAFKAVWSDICETDKA-ITYSDF    565
K.populi_CBS12362               YVGGASQNVSIVRKFSEILGAKEGNYQINLGDACAIGGAFKAVWSDLCETDKA-ITYSDF    565
K.pseudopastoris_CBS9187        YVGGASQNVSIVRKFSEILGAKEGNYQINLGDACAIGGAFKAVWSDLCETDKA-ITYSDF    565
K.kurtzmanii_CBS12817           YVGGASQNVSIVRKFSEVLGAKEGNYQINLGDACAIGGAFKAVWSDLCETEKA-IPYSDF    577
K.phaffii_UWOPS03-328y3         YVGGASQNVSIVRKFSEVLGAKEGNYQINLGDACAIGGAFKAVWSDLCETEKA-IPYSDF    577
K.phaffii_CBS7435               YVGGASQNVSIVRKFSEVLGAKEGNYQINLGDACAIGGAFKAVWSDLCETEKA-IPYSDF    577
K.ulmi_CBS12361                 YVGGASQNVSIVRKFSEILGAKEGNYQINLGDACAIGGAFKAVWSDLCETEKA-ISYSDF    565
K.pastoris_DSMZ70877            YVGGASQNVSIVRKFSEILGAKEGNYQINLGDACAIGGAFKAVWSDLCETKKA-ISYSDF    565
K.pastoris_CBS9178              YVGGASQNVSIVRKFSEILGAKEGNYQINLGDACAIGGAFKAVWSDLCETKKA-ISYSDF    565
K.pastoris_CBS704               YVGGASQNVSIVRKFSEILGAKEGNYQINLGDACAIGGAFKAVWSDLCETKKA-ISYSDF    565
                                ******:* **:**:..:*.  :***:::: :***:***:** **  **:.*  * *.::
```

```
S.stipitis_CBS6054_XKS       INRLFEVSDEMNSFEV-KDKWLEYANGVGMLAKMESELKH*--    623
K.mondaviorum_CBS15017       LTKNFHWKENVKPVEADSSLWLQYAEGVGILSEIEQTLDRDS*    607
K.populi_CBS12362            LSKNFHWKENVKPVEADSSLWLQYAEGVGILSEIEQTLDRDH*    607
K.pseudopastoris_CBS9187     LTKYFHWKENVKPVEADFSLWLQYAEGVGILSEIEQTLDRDL*    607
K.kurtzmanii_CBS12817        LRKNFHWKENVKQVEADSSLWLQYVDGVGILSEIEQTLEK*--    617
K.phaffii_UWOPS03-328y3      LRKNFHWKENVKPVEADSSLWLQYVDGVGILSEIEQTLEK*--    617
K.phaffii_CBS7435            LRKNFHWKENVKPVEADSSLWLQYVDGVGILSEIEQTLEK*--    617
K.ulmi_CBS12361              LSKNFHWKENVKPVEADSSLWLQYVDGVGILSEIEQTLEK*--    605
K.pastoris_DSMZ70877         LSKNFHWKENVKPVEADSSLWLQYVDGVGILSEIEQTLEK*--    605
K.pastoris_CBS9178           LSKNFHWKENVKPVEADSSLWLQYVDGVGILSEIEQTLEK*--    605
K.pastoris_CBS704            LSKNFHWKENVKPVEADSSLWLQYVDGVGILSEIEQTLEK*--    605
                             : : *. .:::: .*.  . **:*.:***:*:::*. *.:
```

Figure S2: Multiple sequence alignments of the *S. stipitis* CBS 6054 xylose pathway enzymes A) xylose reductase (XR), B) xylulose dehydrogenase (XDH) and C) xylulokinase (XKS) with the closest orthologs from all seven *Komagataella species*. Alignments were generated using Clustal Omega [2]. Identical amino acids are marked with an asterisk, a colon indicates residues with highly similar properties and a point indicates residues with weakly similar properties.

```
K.phaffii_PP7435_Chr4-0551      --------------MALAIPQIGFGTGTTWYKYGRESEIDQTLVDALVSALQVGITHLDG    46
K.phaffii_PP7435_Chr2-0714      ------MSRYLQLNNGNKIPHIGFGTWEL----G-----RSQAANVVYHALKAGFRLIDT    45
K.phaffii_Gre3                  ------MATLLKLNNGLKLPQVGLGVWKI----P-----NELTAETVYNAIKQGYRLFDG    45
K.phaffii_Ypr1                  MAVPSNSTKTFKLNNGLSIPAVGLGTWQS----T-----DEEAYNAVIAALKAGYRHIDT    51
                                               .  :* :*:*.              .   :.:  *:: *   :*

K.phaffii_PP7435_Chr4-0551      AECYGTALETRDAIKKA------NIPREKLWITEKYYAGDSSHKSKSKAANPLAALK-EI    99
K.phaffii_PP7435_Chr2-0714      AYLYRNEKEVGEGIERWLDEDPENNKRSDVFYTTKLWDSQFGYENAKR------SIQRAI    99
K.phaffii_Gre3                  AEDYGNEKEVGQGVRRAIDEG--LVKREDLFIVSKLWNNYHHPDNVGK------ALDRTL    97
K.phaffii_Ypr1                  AYCYGNEEPIGKAI----KDS--GVARKDIFITTKLWGTDHTRT--EE------GLDRSL    97
                                *  * .     ..:            *..:: . * :          .      .:.  :

K.phaffii_PP7435_Chr4-0551      LRLEELEYVDLYLLHSPYITKET--------------------HGFTLEEAWGYLEEAQS    139
K.phaffii_PP7435_Chr2-0714      DQVPGLEYIDLLLMHSPNGGPK------------------------VRKETYQAMQEAVD    135
K.phaffii_Gre3                  S-DLGLDYLDLFYIHFPIAFKFVPLEEKYPPAFYCGDGNNFHYEDVPLLDTYRALERLVD    156
K.phaffii_Ypr1                  K-LLGLDYVDLFLMHWPVPMNPNGNHDKFPTLP---DGKRDILFDWNFVDTYREMQKLVA    153
                                     *:*:**  :* *                                :::  ::.

K.phaffii_PP7435_Chr4-0551      LGLAKNIGVSNFTVADLEKILKVA--KVKPQVNQIEYNAFLQDQTPGVVEFSQKNGILIE    197
K.phaffii_PP7435_Chr2-0714      QGIVKQLGVSSWGEAHIKELFSWDGLKYKPVVNQVELSPWCMRE--RLVDFCHKNEILTE    193
K.phaffii_Gre3                  AGRIKSLGVSNFNGALLQDLLRGA--RIKPVALQIEHHPYLVQQ--KLIEYAQSEDIVVV    212
K.phaffii_Ypr1                  SGKTKAIGVSNFSITNLKKLLADPEITIKPVVNQVEIHGYLPQQ--RLLEYAKENDIVLE    211
                                 *  * :***.:  : ::.::       ** . *:*   :   :   ::::.:.: *:

K.phaffii_PP7435_Chr4-0551      AYSPLAPLYKGDKS----IKEVKEF-LDYVYELGKKYGKSDTQVLLKWVLQKGIIPITTS    252
K.phaffii_PP7435_Chr2-0714      AYSPLARGGR--------------FNEQVVKSIAKNKGVTPAQVLLRWSIQKGHIPIPKT    239
K.phaffii_Gre3                  AYSSFGPQSFLELKVNKALTAVSLFEHDVIKKIAQAHNRSAGEVLLRWATQRGLAIIPKS    272
K.phaffii_Ypr1                  AYSPLGSTGA------------PLLKDELVQDLAKKNGISESTLLISWAVWRGIVVLPKS    259
                                *** :.                  :  : : .:.:  . :   :*: *   :*   : .:

K.phaffii_PP7435_Chr4-0551      TKTERVKEFLKIDDFQLEPSE-VEKITTLGAKAPV-VRQYWVPEYSKFD*--    299
K.phaffii_PP7435_Chr2-0714      QTIGRLPENIDVFGFELNDQE----IGQLDH-PNAHDPTDWDVVNVP*----    281
K.phaffii_Gre3                  SKPERLSSNLHINSFDLTK-EDLETISSLDLGLRFNDPWDWDKIPIFA*---    319
K.phaffii_Ypr1                  VTPSRIADNLKIIELCEEDGKKLNELASIRGEKRL-VSPPWDPIVVFNDED*    309
                                 .  *: . :.:  :     :    :  :           *
```

Figure S3: Multiple sequence alignments of the *K. phaffii* CBS 7435 Gre3 homologs. Alignments were generated using Clustal Omega [2]. Identical amino acids are marked with an asterisk, a colon indicates residues with highly similar properties and a point indicates residues with weakly similar properties.

Table S5: Relative abundance of isotopologues of selected metabolites after cultivation on ^{12}C or 1-^{13}C D-xylose, average and standard deviation for n=3 biological replicates.

	^{12}C Xylose		1-^{13}C D-xylose	
**Isotopologue**	**average**	**s**	**average**	**s**
2PG M0	92.6	4.2	69.9	9.1
2PG M1	7.0	3.9	24.0	6.0
2PG M2	0.3	0.5	6.1	3.5
2PG M3	0.0	0.0	0.0	0.0
3PG M0	93.9	5.3	69.7	8.7
3PG M1	5.6	4.7	23.4	6.7
3PG M2	0.5	0.7	6.9	2.1
3PG M3	0.0	0.0	0.0	0.0
6PGA M0	93.6	3.0	91.2	2.1
6PGA M1	3.1	4.4	3.3	4.7
6PGA M2	0.7	1.1	2.0	1.9
6PGA M3	1.9	1.5	3.0	2.1
6PGA M4	0.6	0.4	0.5	0.7
6PGA M5	0.0	0.0	0.0	0.0
6PGA M6	0.0	0.0	0.0	0.0
F6P M0	100.0	0.0	21.6	4.2
F6P M1	0.0	0.0	37.6	4.4
F6P M2	0.0	0.0	25.0	4.2
F6P M3	0.0	0.0	11.9	5.4
F6P M4	0.0	0.0	3.6	1.6
F6P M5	0.0	0.0	0.0	0.0
F6P M6	0.0	0.0	0.4	0.3
G6P M0	98.2	1.0	19.9	2.6
G6P M1	0.4	0.6	39.0	2.9
G6P M2	0.0	0.0	25.2	3.6
G6P M3	1.1	0.6	14.1	0.6
G6P M4	0.2	0.2	1.8	0.7
G6P M5	0.0	0.0	0.0	0.0
G6P M6	0.0	0.0	0.1	0.0
Glc M0	94.7	0.8	36.1	11.3
Glc M1	4.1	0.9	31.5	4.1
Glc M2	0.0	0.0	21.2	4.7
Glc M3	0.5	0.5	10.2	3.7
Glc M4	0.7	1.0	1.0	1.0
Glc M5	0.0	0.0	0.0	0.0
Glc M6	0.0	0.0	0.0	0.1
R5P M0	95.9	0.2	19.7	0.1
R5P M1	4.0	0.2	67.6	1.1
R5P M2	0.0	0.0	11.5	0.5
R5P M3	0.0	0.0	0.8	1.1
R5P M4	0.0	0.0	0.1	0.1
R5P M5	0.0	0.0	0.3	0.2
S7P M0	85.3	9.4	11.2	1.8
S7P M1	7.9	6.1	31.8	1.1
S7P M2	2.0	1.4	40.0	3.9

S7P M3	3.1	1.3	9.8	1.8
S7P M4	1.7	2.4	2.9	1.5
S7P M5	0.0	0.0	2.7	1.0
S7P M6	0.0	0.0	1.6	2.0
S7P M7	0.0	0.0	0.0	0.0

Table S6: List of metabolites and corresponding GC retention times (RT), chemical ionization adducts/fragments evaluated for ^{13}C isotopologue distribution analysis, m/z values of isotopologues extracted and respective mass extraction windows.

Name of derivatized metabolite *	RT (min)	Adduct/ Fragment Evaluated**	Isotopologue* **	m/z	m/z extraction window left / right (ppm)
2PG 4TMS	17.15	$[MH]^+$	M0	475.1583	50 / 50
			M1	476.1617	50 / 50
			M2	477.1650	50 / 50
			M3	478.1684	50 / 50
3PG 4TMS	17.56	$[MH]^+$	M0	475.1583	50 / 50
			M1	476.1617	50 / 50
			M2	477.1650	50 / 50
			M3	478.1684	50 / 50
Glc 5TMS 1EtOx	20.55	$[M-CH_3]^+$	M0	568.2792	50 / 50
			M1	569.2826	50 / 50
			M2	570.2859	50 / 50
			M3	571.2893	50 / 50
			M4	572.2926	50 / 50
			M5	573.2960	50 / 50
			M6	574.2993	50 / 50
R5P 5TMS 1EtOx	24.35	$[M-CH_3]^+$	M0	618.2350	50 / 50
			M1	619.2383	50 / 50
			M2	620.2417	50 / 50
			M3	621.2450	50 / 50
			M4	622.2484	50 / 50
			M5	623.2517	50 / 50
F6P 6TMS 1EtOx	28.59	$[M-CH_3]^+$	M0	720.2851	50 / 50
			M1	721.2884	50 / 50
			M2	722.2918	50 / 50
			M3	723.2951	50 / 50
			M4	724.2985	50 / 50
			M5	725.3018	50 / 50
			M6	726.3052	50 / 50
G6P 6TMS 1EtOx	28.91	$[M-CH_3]^+$	M0	720.2851	50 / 50
			M1	721.2884	50 / 50
			M2	722.2918	50 / 50
			M3	723.2951	50 / 50
			M4	724.2985	50 / 50
			M5	725.3018	50 / 50
			M6	726.3052	50 / 50

6PGA 7TMS	30.02	$[M-CH_3]^+$	M0<br>M1<br>M2<br>M3<br>M4<br>M5<br>M6	765.2773<br>766.2807<br>767.2840<br>768.2874<br>769.2907<br>770.2941<br>771.2974	50 / 50<br>50 / 50<br>50 / 50<br>50 / 50<br>50 / 50<br>50 / 50<br>50 / 50
S7P 7TMS 1EtOx	32.31	$[M-CH_3]^+$	M0<br>M1<br>M2<br>M3<br>M4<br>M5<br>M6<br>M7	822.3351<br>823.3385<br>824.3419<br>825.3452<br>826.3486<br>827.3519<br>828.3553<br>829.3586	50 / 50<br>50 / 50<br>50 / 50<br>50 / 50<br>50 / 50<br>50 / 50<br>50 / 50<br>50 / 50

* names of derivatized metabolites are given specifying the number of trimetylsilyl (TMS) and ethoxyamino groups (EtOx)

** Positive chemical ionization fragments used for data evaluation: $[M+H]^+$: protonated molecular ion, $[M-CH_3]^+$: abstraction of CH_3, $[M+C_2H_5]^+$: addition of C_2H_5

*** number denotes the number of ^{13}C atoms in the backbone

Additional references

1. Ondov BD, Treangen TJ, Melsted P, Mallonee AB, Bergman NH, Koren S, et al. Mash: Fast genome and metagenome distance estimation using MinHash. Genome Biol. 2016;17(1):132.

2. Sievers F, Wilm A, Dineen D, Gibson TJ, Karplus K, Li W, et al. Fast, scalable generation of high-quality protein multiple sequence alignments using Clustal Omega. Mol Syst Biol. 2011;7:539.
